# Maternal and newborn plasma oxytocin levels in response to maternal synthetic oxytocin administration during labour, birth and postpartum – a systematic review with implications for the function of the oxytocinergic system

**DOI:** 10.1186/s12884-022-05221-w

**Published:** 2023-03-02

**Authors:** Sarah Buckley, Kerstin Uvnäs-Moberg, Zada Pajalic, Karolina Luegmair, Anette Ekström-Bergström, Anna Dencker, Claudia Massarotti, Alicja Kotlowska, Leonie Callaway, Sandra Morano, Ibone Olza, Claudia Meier Magistretti

**Affiliations:** 1 Faculty of Medicine, The University of Queensland, Brisbane, Australia; 2 Swedish University of Agricultural Sciences, Uppsala, Sweden; 3 Faculty for Health Sciences, VID Specialized University, Oslo, Norway; 4 Institute for Health Care and Nursing Studies, Martin Luther University Halle-Wittenberg, Halle, Germany; 5 Department of Health Sciences, University West, Trollhättan, Sweden; 6 Institute of Health and Care Sciences, The Sahlgrenska Academy, University of Gothenburg, Göteborg, Sweden; 7 Department of Neurosciences, Rehabilitation, Ophthalmology, Genetics, Maternal and Child Health, University of Genoa, Genoa, Italy; 8 Department of Clinical and Experimental Endocrinology, Faculty of Health Sciences, Medical University of Gdańsk, Gdańsk, Poland; 9 European Institute of Perinatal Mental Health, Madrid, Spain; 10 Institute for Health Policies, Prevention and Health Promotion, Lucerne University of Applied Sciences and Arts, Luzern, Switzerland

**Keywords:** Oxytocin, Maternal oxytocin, Newborn oxytocin, Synthetic oxytocin, Pitocin, Syntocinon, Postpartum oxytocin, Induction of labour, Augmentation of labour

## Abstract

**Background:**

The reproductive hormone oxytocin facilitates labour, birth and postpartum adaptations for women and newborns. Synthetic oxytocin is commonly given to induce or augment labour and to decrease postpartum bleeding.

**Aim:**

To systematically review studies measuring plasma oxytocin levels in women and newborns following maternal administration of synthetic oxytocin during labour, birth and/or postpartum and to consider possible impacts on endogenous oxytocin and related systems.

**Methods:**

Systematic searches of PubMed, CINAHL, PsycInfo and Scopus databases followed PRISMA guidelines, including all peer-reviewed studies in languages understood by the authors. Thirty-five publications met inclusion criteria, including 1373 women and 148 newborns. Studies varied substantially in design and methodology, so classical meta-analysis was not possible. Therefore, results were categorized, analysed and summarised in text and tables.

**Results:**

Infusions of synthetic oxytocin increased maternal plasma oxytocin levels dose-dependently; doubling the infusion rate approximately doubled oxytocin levels. Infusions below 10 milliunits per minute (mU/min) did not raise maternal oxytocin above the range observed in physiological labour. At high intrapartum infusion rates (up to 32 mU/min) maternal plasma oxytocin reached 2–3 times physiological levels.

Postpartum synthetic oxytocin regimens used comparatively higher doses with shorter duration compared to labour, giving greater but transient maternal oxytocin elevations. Total postpartum dose was comparable to total intrapartum dose following vaginal birth, but post-caesarean dosages were higher.

Newborn oxytocin levels were higher in the umbilical artery vs. umbilical vein, and both were higher than maternal plasma levels, implying substantial fetal oxytocin production in labour. Newborn oxytocin levels were not further elevated following maternal intrapartum synthetic oxytocin, suggesting that synthetic oxytocin at clinical doses does not cross from mother to fetus.

**Conclusions:**

Synthetic oxytocin infusion during labour increased maternal plasma oxytocin levels 2–3-fold at the highest doses and was not associated with neonatal plasma oxytocin elevations. Therefore, direct effects from synthetic oxytocin transfer to maternal brain or fetus are unlikely. However, infusions of synthetic oxytocin in labour change uterine contraction patterns. This may influence uterine blood flow and maternal autonomic nervous system activity, potentially harming the fetus and increasing maternal pain and stress.

**Supplementary Information:**

The online version contains supplementary material available at 10.1186/s12884-022-05221-w.

## Background

Oxytocin is of critical importance for labour and birth through its stimulatory effects on uterine contractions [1]. Oxytocin is also involved in bonding, maternal caregiving, lactation and stress regulation, among other biological effects [2]. Synthetic oxytocin (Syntocinon, Pitocin, exogenous oxytocin) has an identical chemical structure to endogenous oxytocin and is widely administered to women to induce or augment labour, and to prevent or treat postpartum haemorrhage.

Endogenous oxytocin is produced by neurons within the supra-optic and paraventricular nuclei (SON and PVN) in the hypothalamus and transferred to the posterior pituitary gland for release into the circulation to the periphery. Peripheral physiological effects of endogenous oxytocin include facilitating the uterine contractions of labour and birth and the milk-ejection (‘let down’) reflex of lactation. In addition, oxytocin from the SON and PVN reaches and impacts widespread areas in the brain via oxytocinergic nerves and axon collaterals, and by dendritic release from the SON and PVN. In this way oxytocin can exert integrated psychophysiological effects [3, 4].

Maternal oxytocin levels rise gradually during pregnancy in response to rising levels of estrogen. Estrogen also increases the numbers and function (binding) of uterine oxytocin receptors in preparation for labour, birth and postpartum transitions [1]. As labour commences, pulses of oxytocin are released from the pituitary. These pulses increase in frequency, duration and amplitude, reaching a maximal frequency of three pulses per ten minutes [5]. These oxytocin peaks are preceded by brief (milliseconds) periods of electrical activity in the oxytocin neurons in the hypothalamus [6].

Oxytocin is also produced within the uterine decidua and other local tissues, and there are oxytocin receptors within these tissues [7, 8]. During labour, this paracrine oxytocin causes an increase in decidual prostaglandin production. Local prostaglandins contribute to myometrial contractions and likely cervical changes and may give local positive feedback to oxytocin pro-contractile effects [7, 9]. Findings from transgenic animal studies suggest that other processes and pathways to parturition may exist outside of the classical oxytocin system, reflecting the critical role of parturition in mammalian survival [10, 11].

The autonomic nervous system (ANS) also contributes to the control of uterine contractions and labour progress. The uterus is innervated by both branches of the ANS: the parasympathetic nervous system (PSNS) and the sympathetic nervous system (SNS).

Parasympathetic pathways involve oxytocin nerves that originate in oxytocin-producing areas of the PVN and reach the uterus via cholinergic neurons of the PSNS ganglia in the lumbosacral region [12, 13]. Stimulation of outgoing (motor, efferent) PSNS nerve pathways from the brain causes uterine contractions and increases blood supply to the uterus [14]. In contrast, stimulation of SNS efferent nerves can cause long-lasting, painful and/or ineffective contractions and reduce uterine blood flow [14].

Labour processes are also controlled centrally by incoming (sensory, afferent) ANS nerves, which transmit information about the physiological state of the cervix, vagina and uterine muscles to regulatory centres in the brain. This sensory information helps to regulate oxytocin release during labour [15].

Oxytocin release is further promoted in labour by the Ferguson reflex, a positive feedback cycle that is stimulated by sensory input from the pressure of the baby’s head on the cervix, caused by uterine contractions [16]. This sensory input, transmitted by PSNS sensory nerves via spinal cord pathways, triggers the release of oxytocin from the posterior pituitary into the circulation [1]. Oxytocin release further strengthens uterine contractions, and therefore pressure from the fetal head on the cervix, fuelling this positive feed-back cycle [1].

**Fig. 1:**
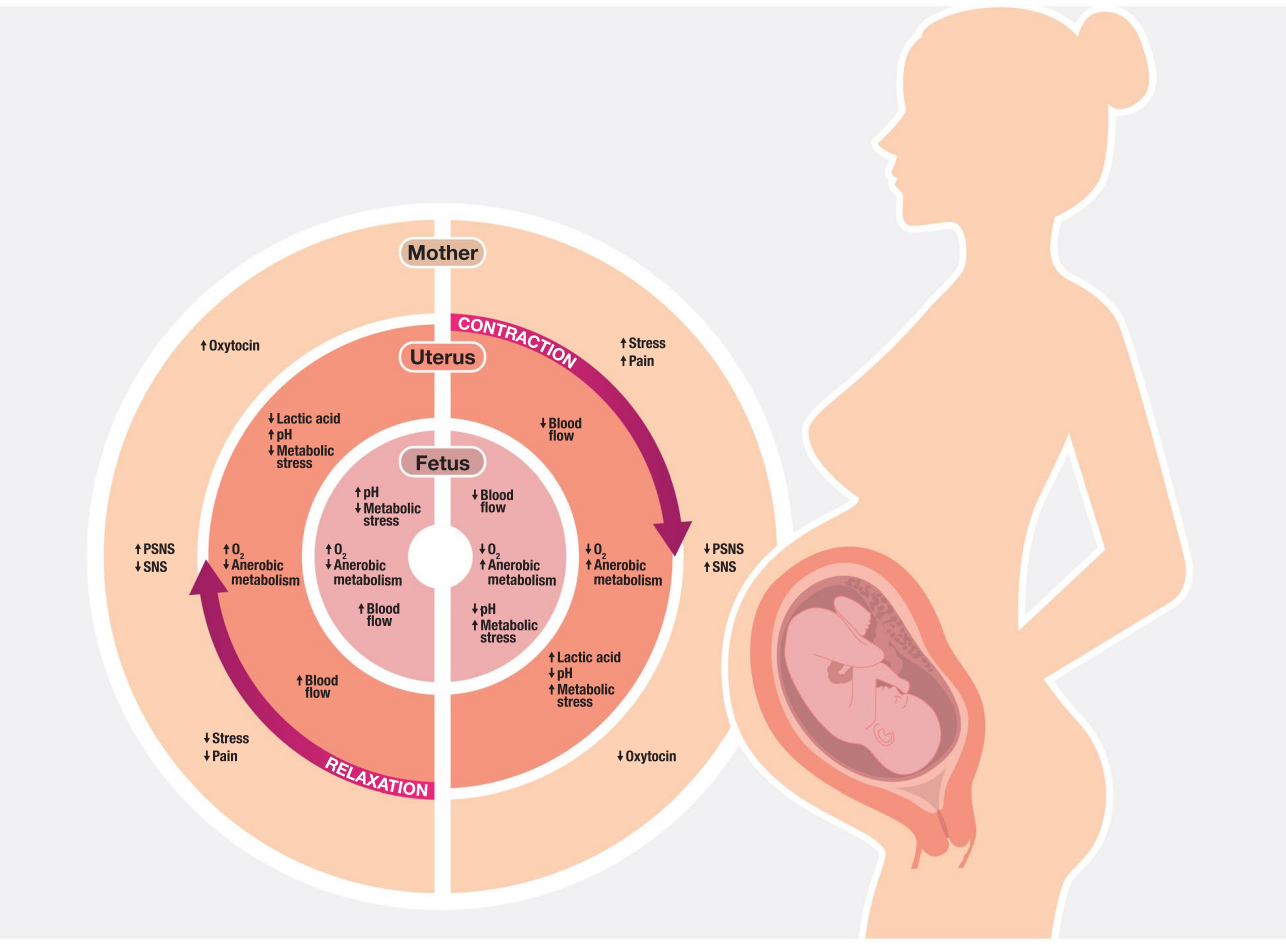
Uterine contraction and relaxation: metabolic, autonomic and haemodynamic effects for mother, uterus, and fetus. Note: Equal lengths of contraction and relaxation are for illustration only. In labour the period of contraction is relatively shorter, with a relatively longer period of relaxation that allows full replenishment of blood supply to the uterus and fetus, as shown in the figure. As labour progresses, contractions become stronger and more frequent with relatively shorter periods of relaxation in between. Infusions of synthetic oxytocin cause stronger and more frequent contractions, which further shortens the relative time for replenishment in uterine tissues. This may increase maternal pain and stress and reduce fetal blood flow. In this way, administration of synthetic oxytocin may exaggerate maternal metabolic and autonomic consequences and fetal blood flow reductions induced by the contractions of physiological birth, as illustrated. Abbreviations: O_2:_ oxygen; pH: measure of acidity; PSNS: parasympathetic nervous system; SNS: sympathetic nervous system (Figure 1 copyright S Buckley and K Uvnäs-Moberg, 2023)

Oxytocin levels during labour may also be influenced by local conditions in the uterine tissues. When the uterine muscles contract, local pressures temporarily occlude the blood supply, creating relatively low oxygen levels in the muscle. The resulting anaerobic metabolism produces lactic acid and increased acidity (lower pH), which inhibits intramuscular calcium channels, weakening or even stopping contractions. As the uterine contraction subsides, blood flow is restored, with increased oxygenation and removal of acid metabolites. These metabolic changes provide feedback inhibition for the current uterine contraction and subsequently prepare the uterine muscles for the next contraction. This model is illustrated in Fig. 1 and well described by Wray and Wiberg-Itzel [17–19].

In addition, autonomic sensory nerves originating in the myometrium are involved in this metabolic feedback mechanism. During contractions, SNS nerves are activated in response to the local metabolic stresses and induce maternal pain and stress via central actions. In addition, as the ANS balance shifts towards the SNS, oxytocin release is decreased [15]. There may also be a functional decrease in oxytocin receptor function as part of this feedback inhibition, although this has not been studied.

As the contraction and the contraction-related metabolic stresses subside, signalling in the SNS decreases and the inhibition of oxytocin release is withdrawn. The balance in the ANS shifts from the SNS back towards the PSNS, with rising oxytocin levels further promoting the next uterine contraction [17–19]. (See Fig. 1). Oxytocin receptor function may also be reinstated. This model is also supported by the findings of variations in oxytocin levels during the contractions of physiological labour [20].

Oxytocin is elevated not only in the circulation but also within the maternal brain from labour to postpartum. Oxytocin elevations counteract stress, fear and pain, and induce positive feelings in birthing women [21]. Oxytocin also facilitates beneficial maternal adaptations, including the activation of brain reward centres that facilitate maternal-newborn bonding and caretaking [1, 21] Oxytocin elevations in labour may also sensitise maternal skin, so that the new mother maximally releases oxytocin during skin-to-skin contact with her newborn, and promote the initiation of lactation [1, 2].

The fetus also produces oxytocin, which is released during labour and birth into both the brain and circulation. Fetal oxytocin release may be promoted by skin stimulation from uterine contractions and also by the physiological stresses of labour [22]. Oxytocin provides beneficial analgesic, antioxidant and anti-inflammatory effects for the fetus and newborn [23, 24].

Other physiological and hormonal processes also provide substantial protection for the fetus from contraction-related reductions in blood and oxygen during labour. The inevitable hypoxia of labour, along with increasing pressure on the fetal head, triggers the release of very high levels of adrenaline and noradrenaline. This ‘fetal catecholamine surge’ prioritises essential blood supply to the heart and brain and promotes anerobic glycolysis, among other fetal adaptations [25–28].

This model of oxytocin-associated metabolic and physiological effects with contractions is also valid for the effects of synthetic oxytocin on contractions. With synthetic oxytocin, contractions are stronger and also more frequent, which makes the relative duration of contractions vs. relaxation longer [29–31]. This exaggerates the maternal metabolic and autonomic and fetal haemodynamic effects caused by contractions, as described in Fig. 1. Therefore, synthetic oxytocin may be linked to more negative consequences than physiological labour for mother and baby, especially when high infusion rates cause significantly stronger and more frequent contractions.

It is estimated that up to half of women giving birth in institutionalised maternity care systems, including in low- and middle-income countries, will receive synthetic oxytocin for labour induction or augmentation [32–34]. In addition, routine administration of synthetic oxytocin is recommended to prevent postpartum haemorrhage after vaginal birth, either by intramuscular injection or intravenous administration [35, 36]. Synthetic oxytocin can also be administered by postpartum intravenous infusion to treat haemorrhage following vaginal birth or routinely after caesarean section.

With this widespread use of synthetic oxytocin in labour, birth and postpartum, it is important to review the data on oxytocin levels in connection with perinatal synthetic oxytocin administration for several reasons.

Data on maternal and newborn levels of oxytocin in response to administration of synthetic oxytocin are not readily available and there is no review summarising this data. A primary aim of this review was to summarise this data, making it understandable and accessible for clinicians and other researchers.

Another aim of this study was to illustrate that administration of synthetic oxytocin by intravenous infusion follows the expected pharmacological rules. Plasma oxytocin levels would be expected to rise in a dose-dependent way, have a well-defined half-life and achieve steady state levels after a defined period of time, analogous to what is observed after administration of other drugs. An available summary of this data might aid clinicians to ascertain the appropriate infusion rates of synthetic oxytocin during labour and postpartum and reduce the chances of adverse effects.

High infusion rates of synthetic oxytocin might result in supra-physiological oxytocin levels, which could have biological impacts for women and babies in labour. A further aim was therefore to provide data that might assist with assessing the likelihood of potential side-effects, short- or longer-term, for women administered synthetic oxytocin in the perinatal period and their offspring.

## Methods

The aim of this study was to systematically review the existing literature on maternal and newborn plasma oxytocin levels following maternal synthetic oxytocin administration during labour, birth and/or postpartum and to consider possible implications for women and offspring.

A systematic literature search was undertaken according to the PRISMA statement with the aim of summarising existing research regarding the effect of maternal administration of synthetic oxytocin on maternal and newborn plasma oxytocin levels [37].

### Selection of studies and eligibility

An a priori protocol was designed with the aim, procedure and inclusion criteria. We included studies of women in labour, birth and/or postpartum and their newborns (participants) who were exposed to maternal administration of synthetic oxytocin (intervention) and who had at least one post-intervention measurement of plasma oxytocin levels (outcome), allowing comparison with pre-intervention levels or with controls who did not receive synthetic oxytocin, where such data was available (comparison).

All types of peer-reviewed articles reporting original research written in any language understood by the research team (English, German, Spanish, French, Swedish) were considered, with any date of publication up to June 14, 2022. In addition, findings were included from one Swedish PhD thesis that included a rigorous peer review as part of the examination process, and from several publications in Japanese, including one with unique data that was professionally translated. Inclusion criteria are listed in Table 1.

**Table 1:** Inclusion and exclusion criteria

INCLUSION: **Population:** Women administered synthetic oxytocin during labour, birth, or postpartum and their newborns **Outcome**: Maternal, fetal and/or or newborn plasma oxytocin levels during labour, birth, or postpartum, measured by any technique **Included articles:** All types of peer-reviewed original research studies reporting at least one measurement of plasma oxytocin in relation to synthetic oxytocin administration No limitation in years **Languages:** Publications in languages understood by any member of the research team including English, German Spanish, French, and Polish. (Translation was considered for any highly relevant publications with unique data) EXCLUSION: Reports, abstracts, study protocols, conference proceedings, case reports Languages not understood by any member in the research team	

We included only studies that measured plasma oxytocin levels and not salivary or urine oxytocin levels, as such measurements have not been shown to accurately mirror plasma oxytocin levels or physiological patterns [38–41].

### Search strategy and screening

The search strings were created by SB and AEB, together with librarians from the University of Queensland, Australia, and the University of Skövde, Sweden. Searches were performed in September 2017 in the following databases: PubMed, Scopus, CINAHL (Cumulative Index of Nursing and Allied Health Literature), and PsycInfo. Additional literature searches using the same search strings were completed in March 2020 and June 2022. The search terms comprise synonyms and database-specific terms for oxytocin AND levels AND blood/plasma AND labour/birth/breastfeeding/interventions/newborns. The full search strings are available in Additional file 1.

In total, 3847 articles were identified via database searches (PubMed *n* = 1598, Scopus *n* = 1769, CINAHL *n* = 247, and PsycInfo *n* = 233). The reference lists of all eligible publications were also hand searched and eight additional articles were found (total 3855). After the removal of 613 duplicates, the remaining 3242 articles were screened on title and abstract and 2914 were excluded. After the full-text screening of the remaining 328 articles, 35 articles were identified that met the inclusion criteria. These 35 publications are based on 31 clinical studies, as four publications reported findings from other included studies.

**Fig. 2:**
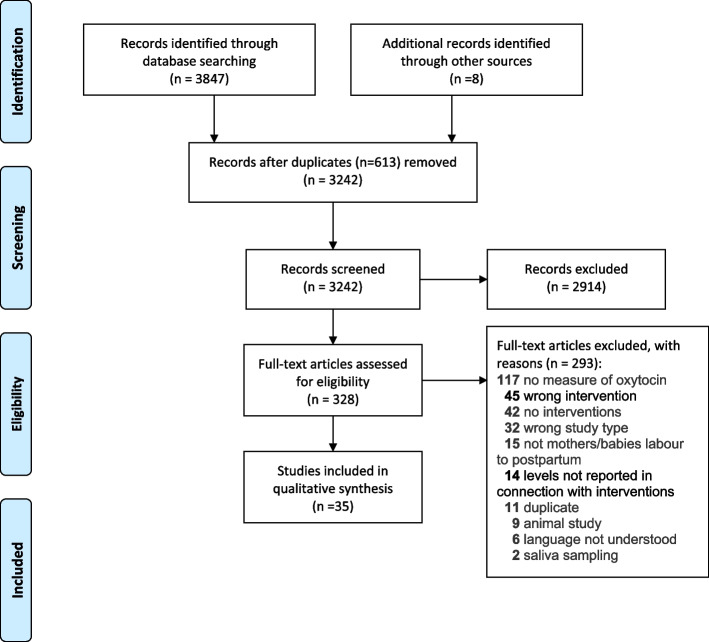
Selection process (Prisma)

At each stage, articles were screened by at least two authors, working independently in pairs, based on the inclusion and exclusion criteria (Table 1). Initial title and abstract screening were performed using the Covidence© online platform by AEB, CM, GDB, KL, KUM, SB and ZP. In case inclusion was unclear, a third expert author (KUM) was involved. Subsequent screening, hand searches, full-text review, final inclusion, and data extraction were done by SB and KUM. The selection process is illustrated in Fig. 2, based on the PRISMA (Preferred Reporting Items for Systematic reviews and Meta-Analyses) protocol, including reasons for exclusion at full-text screening [37].

### Data extraction and analysis

Altogether, 1373 women and 148 newborns participated in studies reported in the 35 publications summarised in this paper. The included publications are listed in Table 2, ‘Characteristics of included studies,’ together with background data including the type of interventions and the numbers of participants, including women and newborns without exposure to synthetic oxytocin (controls).

**Table 2:** Characteristics of included studies

Authors, year, title, journal [Citation number]	Intervention studied W: number of women participants included in this review NB: numbers of newborn participants included in this review (Participants also reported in another included study as noted)				Comments
	Synthetic oxytocin (SOT) in labour	Synthetic oxytocin postpartum	Synthetic oxytocin with epidural	Controls	
**Amico JA.; Seitchik J.; Robinson AG. (1984). Studies of oxytocin in plasma of women during hypocontractile labor.** The Journal of Clinical Endocrinology and Metabolism Feb 1984;58(2):274–9 [42]	(11 W)				Data from this study is also published in Seitchik (1984)
**Amico JA.; Ervin MG.; Finn FM.; Leake RD.; Fisher DA.; Robinson AG. (1986). The plasma of pregnant women contains a novel oxytocin-vasotocin-like peptide**. Metabolism: clinical and experimental Jul 1986;35(7):596–601 [43]	4 W				
**Arai T. (1980). The significance of plasma oxytocin in pregnancy and at parturition.** Acta Obstet. Gynaecol. Jpn. 1980;32(12):2017–**2026** [20]	14 W			16 W	Publication in Japanese Data obtained from full article professionally translated into English
**Dawood MY.; Wang CF.; Gupta R.; Fuchs F. Fetal contribution to oxytocin in human labor (1978).** Obstetrics and Gynecology Aug 1978;52(2):205–9 [44]	7NB			26NB	Women received SOT by buccal (intra-oral) and/or intravenous routes
**Dawood, M.; Ylikorkala, O.; Fuchs, F. (1980). Plasma oxytocin levels and disappearance rate after buccal Pitocin**. Am. J. Obstet. Gynecol. 1980;138(1):20–24 [45]	9 W				
**De Tina, A.; Juang, J.; McElrath, T.F.; Baty, J.D.; Palanisamy, A. (2019)**. **Oxytocin and Oxytocinase in the Obese and Nonobese Parturients during Induction and Augmentation of Labor**. AJP Rep. 2019;9(2):E177-E184 [46]	18 W				Data from the 18/25 non-obese women with complete data are included in this review Similar results reported in obese group (*n* = 12/25 with complete data)
**Ende HB; Soens MA; Nandi M; Strichartz GR; Schreiber KL (2019)**. **Association of Interindividual Variation in Plasma Oxytocin With Postcaesarean Incisional Pain.** Anesth Analg Oct 2019;129(4):e118-e121 [47]		18 W			Post-caesarean
**Erickson EN; Carter CS; Emeis CL. Oxytocin, Vasopressin and Prolactin in New Breastfeeding Mothers: Relationship to Clinical Characteristics and Infant Weight Loss**. J Hum Lact Feb 2020;36(1):136–145 [48]	11 W	(33/46 W)		35 W	33/46 women received SOT postpartum as well as intrapartum Oxytocin data for women with and without SOT exposure was combined
**Fuchs AR.; Husslein P.; Sumulong L.; Fuchs F. The origin of circulating 13,14-dihydro-15-keto-prostaglandin F2 alpha during delivery (1982).** Prostaglandins Nov 1982;24(5):715–22 [49]		10 W		10 W	Data from this study is also published in Husslein (1983a)
**Fuchs, A.-R.; Goeschen, K.; Husslein, P.; Rasmussen, A.B.; Fuchs, F. (1983). Oxytocin and the initiation of human parturition. III. Plasma concentrations of oxytocin and 13,14-dihydro-15-keto-prostaglandin F2α in spontaneous and oxytocin-induced labor at term.** Am. J. Obstet. Gynecol. 1983;147(5):497–502 [50]	15 W			17 W	
**Fuchs AR.; Romero R.; Keefe D.; Parra M.; Oyarzun E.; Behnke E. (1991). Oxytocin secretion and human parturition: pulse frequency and duration increase during spontaneous labor in women.** American Journal of Obstetrics and Gynecology Nov 1991;165(5 Pt 1):1515–23 [5]	18 W			32W	
**Furuya K.; Nagata I.; Imaizumi E.; Hirata J.; Kato K. (1988). [Fundamental studies on the measurement of plasma concentration of oxytocin during perinatal period]**. Nihon Sanka Fujinka Gakkai Zasshi Nov 1988;40(11):1685–92 [51]	5 W				Publication in Japanese Data obtained from English abstract and tables
**Gibbens D.; Boyd NR.; Crocker S.; Baumber S.; Chard T. (1972) The circulating levels of oxytocin following intravenous and intramuscular administration of Syntometrine**. J Obstet Gynaecol Br Commonw. 1972;79(7):644–6 [52]		26 W			
**Gu V.; Feeley N.; Gold I.; Hayton B.; Robins S.; Mackinnon A.; Samuel S.; Carter CS.; Zelkowitz P. (2016)**. **Intrapartum Synthetic Oxytocin and Its Effects on Maternal Well-Being at 2 Months Postpartum**. Birth (Berkeley, Calif.) Mar 2016;43(1):28–35 [53]	386 W With and without SOT intrapartum and/or postpartum			(29 W)	Oxytocin data for women with and without SOT exposure was combined Oxytocin data for women with intrapartum and postpartum SOT exposure was combined
**Handlin, L.; Jonas, W.; Petersson, M.; Ejdebäck, M.; Ransjö-Arvidson, A.-B.; Nissen, E.; Uvnäs-Moberg, K. (2009)**. **Effects of sucking and skin-to-skin contact on maternal ACTH and cortisol levels during the second day postpartum-influence of epidural analgesia and oxytocin in the perinatal period**. Breastfeeding Med. 2009;4(4):207–220 [54]	9 W	14 W	14 W	20 W	Data from this study is also published in Jonas (2009) Small discrepancies in numbers reported in this publication vs. Jonas (2009) Other data from this study regarding blood pressure, cortisol levels and personality changes in relation to interventions is published in separate articles
**Husslein P.; Fuchs AR.; Fuchs F. (1983a)**. **[Oxytocin- and prostaglandin plasma concentrations before and after spontaneous labor: evidence of involvement of prostaglandins in the mechanism of placental separation]**. Wiener klinische Wochenschrift May 1983;95(11):367–71 [55]		(10 W)		(10 W)	Publication in German Data obtained from English abstract and tables and author (KUM) translation Data from this study is also published in Fuchs et al. (1982)
**Husslein P.; Kofler E.; Rasmussen AB.; Sumulong L.; Fuchs AR.; Fuchs F. (1983b)**. **Oxytocin and the initiation of human parturition. IV. Plasma concentrations of oxytocin and 13,14-dihydro-15-keto-prostaglandin F2 alpha during induction of labor by artificial rupture of the membranes**. American journal of obstetrics and gynecology Nov 1983;147(5):503–7 [56]	6 W			16W	
**Jonas K.; Johansson LM.; Nissen E.; Ejdebäck M.; Ransjö-Arvidson AB.; Uvnäs-Moberg K. (2009)**. **Effects of intrapartum oxytocin administration and epidural analgesia on the concentration of plasma oxytocin and prolactin, in response to suckling during the second day postpartum**. Breastfeeding medicine: the official journal of the Academy of Breastfeeding Medicine Jun 2009;4(2):71–82 [57]	(8 W)	(13 W)	(14 W)	(20 W)	Data from this study is also published in Handlin (2009) Other data from this study regarding blood pressure, cortisol levels and personality changes in relation to interventions is published in separate articles
**Otsuki Y.; Tanizawa O.; Yamaji K.; Fujita M.; Kurachi K. (1983)**. **Feto-maternal plasma oxytocin levels in normal and anencephalic pregnancies**. Acta obstetricia et gynecologica Scandinavica 1983;62(3):235–7 [58]	5 W 5NB			6 W 6NB	Also included 4 women and their newborns with anencephaly
**Padayachi, T.; Norman, R.J.; Dhavaraj, K.; Kemp, M.; Joubert, SM. (1988)**. **Serial oxytocin levels in amniotic fluid and maternal plasma during normal and induced labour**. BJOG Int. J. Obstet. Gynaecol. 1988;95(9):888–893 [59]	8 W 8NB			13 W 13NB	
**Patient C.; Davison JM.; Charlton L.; Baylis PH.; Thornton S. (1999)**. **The effect of labour and maternal oxytocin infusion on fetal plasma oxytocin concentration**. British journal of obstetrics and gynaecology Dec 1999;106(12):1311–3 [60]	10NB			15NB	
**Perry RL.; Satin AJ.; Barth WH.; Valtier S.; Cody JT.; Hankins GD. (1996)**. **The pharmacokinetics of oxytocin as they apply to labor induction**. American journal of obstetrics and gynecology May 1996;174(5):1590–3 [61]	10 W				
**Pochard, J.L.; Lutz-Bucher, B. (1986)**. **Vasopressin and oxytocin levels in human neonates. Relationships with the evolution of labour and beta-endorphins**. Acta Paediatr. Scand. 1986;75(5):774–778 [62]	6 W 6NB			21 W 21NB	Also included 10 women and their newborns with fetal distress in labour
**Prevost M.; Zelkowitz P.; Tulandi T.; Hayton B.; Feeley N.; Carter CS.; Joseph L.; Pournajafi-Nazarloo H.; Yong Ping E.; Abenhaim H.; Gold I. (2014)**. Oxytocin in pregnancy and the postpartum: relations to labor and its management. Frontiers in public health 2014;2(1):1–9 [63]	272 W With and without SOT intrapartum				Oxytocin data for women with and without SOT exposure was combined
**Risberg A.; Sjöquist M.; Wedenberg K.; Olsson U.; Larsson A. (2015)**. **Water balance during parturition and early puerperium: A prospective open trial.** Clinical biochemistry Sep 2015;48(13–14):837–42 [64]	12 W	(25 W)		30 W	Included additional 9 women with IV fluids and no SOT
**Seitchik J.; Amico J.; Robinson AG.; Castillo M. (1984)**. **Oxytocin augmentation of dysfunctional labor. IV. Oxytocin pharmacokinetics**. American journal of obstetrics and gynecology Oct 1984;150(3):225–8 [65]	11 W				Data from this study is also published in Amico (1984)
**Seitchik J.; Amico JA.; Castillo M. (1985)**. **Oxytocin augmentation of dysfunctional labor. V. An alternative oxytocin regimen**. American journal of obstetrics and gynecology Mar 1985;151(6):757–61 [66]	10 W				
**Sellers SM.; Hodgson HT.; Mountford LA.; Mitchell MD.; Anderson AB.; Turnbull AC. (1981)**. **Is oxytocin involved in parturition?** British journal of obstetrics and gynaecology Jul 1981;88(7):725–9 [67]		9NB		8NB	
**Takahashi, Y.; Uvnäs-Moberg, K.; Nissen, E.; Lidfors, L.; Ransjö-Arvidson, A. B.; Jonas, W. (2021). Epidural Analgesia With or Without Oxytocin, but Not Oxytocin Alone, Administered During Birth Disturbs Infant Pre-feeding and Sucking Behaviors and Maternal Oxytocin Levels in Connection With a Breastfeed Two Days Later.** Front Neurosci 2021;15:673184 [68]	(5 W)	(8 W)	(10 W)	(13 W)	This study is a reanalysis of published in Handlin (2009) and Jonas (2009) Other data from this study regarding blood pressure, cortisol levels and personality changes in relation to interventions is published in separate articles [updated search 2022 Rev1]
**Thornton S.; Davison JM.; Baylis PH. (1988). Plasma oxytocin during third stage of labour: comparison of natural and active management.** BMJ (Clinical research ed.) Jul 1988;297(6642):167–9 [69]		10 W		15 W	
**Thornton S.; Davison JM.; Baylis PH. (1990). Effect of human pregnancy on metabolic clearance rate of oxytocin.** The American journal of physiology Jul 1990;259(1 Pt 2):R21–4 [70]	10 W	10 W			Experimental study of SOT infusions to women at 8–10 weeks postpartum
**Thornton S.; Davison JM.; Baylis PH. (1992). Plasma oxytocin during the first and second stages of spontaneous human labour.** Acta endocrinologica May 1992;126(5):425–9 [71]	5 W			7 W	
**Velandia, M (2012). Parent-infant Skin-to-Skin Contact Studies. (PhD Thesis)** Stockholm, Sweden: Karolinska Institute [72].		19 W		15 W	Post-caesarean
**Yamaguchi, ET.; Cardoso, MM.; Torres, ML.; Nascimento, RC.; Ribeiro, MC.; Frerichs, E.; Payen, D. (2011). Serum oxytocin concentrations in elective caesarean delivery: A randomized comparison of three infusion regimens.** Int. J. Obstet. Anesth. 2011;20(3):224–228 [73]		30 W			Post-caesarean
**Yuksel, B.; Ital, I.; Balaban, O.; Kocak, E.; Seven, A.; Kucur, SK.; Erbakirci, M.; Keskin, N.** Immediate breastfeeding and skin-to-skin contact during cesarean section decreases maternal oxidative stress, a prospective randomized case-controlled study. J. Matern.-Fetal Neonatal Med. 2016;29(16):2691–2696 [74]		90 W			Post-caesarean

*Abbreviations*: *NB* numbers of newborn participants included in this review, *SOT* synthetic oxytocin, *W* number of women participants included in this review

It was not possible to perform a meta-analysis because the study designs varied substantially in relation to indications for administration of oxytocin; dose regimens and timing; presence of a control group; timing, frequency and techniques of blood sampling; assay types and assay sensitivities. We therefore extracted data from each publication. Text summaries were formulated, and the extracted data was also summarised in separate tables for maternal and newborn oxytocin levels (Tables 3 and 4 respectively.)

### Quality assessment considerations

We ensured that the quality of the data was as high as possible through strict inclusion criteria. We carefully subdivided the data into groups of similar study designs. We also paid close attention to techniques and methodologies that might influence the results, including types of assays and assay sensitivities. The strengths and limitations of each study are further discussed in the tables and text.

It is noteworthy that many of the included studies were published more than 20 years ago. However, most of these older studies are of very high quality and assess plasma oxytocin levels with frequent and multiple sampling and high-quality assays, giving comprehensive and reliable data. In contrast, some of the more recent studies were limited by technical, practical, and other considerations, and generally included fewer samples.

Endogenous and synthetic oxytocin are biochemically identical and therefore the same techniques can be used to measure both synthetic and endogenous oxytocin in plasma.

An important quality assessment consideration is the type of assay used to measure plasma oxytocin levels. Radioimmunoassay (RIA) is the gold standard but is expensive and requires radioactive material. More recent studies have more often used Enzyme-Linked Immunosorbent Assay/Immunoassay (ELISA, EIA). Oxytocin levels and effect patterns measured with ELISA can differ from those obtained by RIA, with values up to 10–100 fold higher, especially without sample extraction prior to analysis. In addition, ELISA is less specific, as the high levels may reflect not only oxytocin but also fragments or metabolites of oxytocin [75, 76]. It is particularly relevant to this review that some studies using ELISA have not found the physiological oxytocin elevations that occur with advancing gestation or during breastfeeding, which are clearly seen with RIA (See Uvnas Moberg 2019, 2020 for systematic reviews) [1, 2]. The type of assay used in each study is listed in the tables, and the type of assay was also considered when interpreting the data.

Oxytocin sampling techniques are another critical quality assessment consideration. The short pulses of oxytocin that are released during labour and birth are best detected with frequent, rapid sampling at short intervals. However, it is not simple to take multiple blood samples from labouring women, especially in late labour. Therefore, samples tend to cluster at the start of labour, with fewer samples at the end. Sampling frequency and timing is listed in each table and is also considered in the interpretation of the data. A more detailed consideration of plasma oxytocin sampling techniques is presented in a previously published review [1].

The included papers also used a variety of measures of plasma oxytocin levels, including microunits per millilitre (μU/mL), picograms per millilitre (pg/mL) and picomoles per litre (pmol/L or pM). To facilitate comparisons between studies, we converted all results to picograms per millilitre (pg/mL), which is the most commonly-used measure. Data is also listed in the original units, and conversions are provided in the table legends. (See also Uvnäs-Moberg (2019) for a comprehensive conversion table [1].)

## Results

The characteristics of the 35 included studies are listed in Table 2: ‘Characteristics of included studies.’ All other details including results are listed in Table 3: ‘Maternal synthetic oxytocin administration: maternal plasma oxytocin levels’ and Table 4: ‘Maternal synthetic oxytocin administration: newborn cord blood and maternal plasma oxytocin levels.’

**Table 3:** Maternal synthetic oxytocin administration: maternal plasma oxytocin levels

Publication details	Number of participants Indication	Dose regimen in mU unless otherwise stated (converted from original units) Total or maximum dose Duration Sampling regimen	Maternal plasma oxytocin levels, pg/mL, mean ± SEM or SD (converted from original units) Important findings	Oxytocin assay Comments Other reported data
**Single dose IV bolus before labour**				
**Fuchs et. al. (1991)** Oxytocin secretion and human parturition: pulse frequency and duration increase during spontaneous labor in women	**18 women** Experimental study of bolus SOT in late pregnancy	**Dose regimen:** Single IV bolus of 2, 4, 8 or 16 mU **Total dose:** 2, 4, 8 or 16 mU **Sampling regimen:** Baseline and at 1, 2, 3, 4, 5 and 10 min	**Results:** *(Estimated from Fig. 5)* Range, pg/mL (μU/mL) ***Basal (late pregnancy)*** 0.5–0.7 pg/mL (0.3–0.4 μU/mL) ***With SOT infusion*** 0.8–1.7 pg/mL (0.5–1.0) peak **Important findings:** Dose-dependent rise at 1–2 min at doses 4 mU or higher Decline to approx. Basal over 10 min	**Assay:** RIA **Comments:** Levels similar to spontaneous pre-labour pulses, as measured in this study **Other reported data:** Contraction pattern and oxytocin levels Number of contractions following the bolus correlated with mean oxytocin peak levels
**Intravenous infusion for labour induction or augmentation: studies with dose-response data and findings**				
**Amico et. al. (1984)** Studies of oxytocin in plasma of women during hypocontractile labor	**11 women** Labour augmentation with SOT	**Dose regimen**: IV infusion 1 mU/min increased by 1 mU/min every 40 min until ‘adequate contractility’ up to 4 mU/min **Maximum dose:** Mean 3.4 ± 0.7 mU/min Range 1–8 mU/min **Sampling regimen:** Baseline and every 20 min until 60–100 min after final infusion rate increase	**Results:** *(Estimated from Fig. 4)* Mean ± SEM, pg/mL (μU/mL) ***Basal (in labour)*** 1.7 ± 0.52 pg/mL (1.0 ± 0.31) μU/ml) ***With SOT infusion*** 2.7 ± 0.5 (1.6 ± 0.3) at 1 mU/min 3.8 ± 0.7 (2.3 ± 0.4) at 2 mU/min 4.8 ± 1.1 (2.9 ± 0.7) at 3 mU/min 6.3 ± 1.5 (3.8 ± 0.9) at 4 mU/min **Important findings:** Steady-state oxytocin levels reached 40 min after SOT dose Linear dose-dependent rise (r^2^ = 0.99)	**Assay:** RIA RIA assay giving very low basal levels **Comments:** Doubling infusion rate approx doubled oxytocin levels **Other reported data:** ***Oxytocin metabolic clearance rate (MCR)*** 11–32.5 (mean 17.3) mL/kg/min ***SOT administered to 4 men at 125 mU/min*** 1.3 pg/mL (0.76 μU/mL) basal 188 (112.5) at 60 min Linear dose-dependent rise Steady state oxytocin levels at 30 min Oxytocin MCR 25–34 (mean 17.6) mL/kg/min ***SOT half-life: 12.3–17.4 min*** Data from this study also published in Seitchik et al. (1984)
**Amico et. al. (1986)** The plasma of pregnant women contains a novel oxytocin-vasotocin-like peptide.	**4 women** Labour augmentation with SOT	**Dose regimen:** IV infusion 1 mU/min increased 1 mU/min every 40 minutes **Maximum dose:** 4 mU/min **Sampling regimen:** Baseline, every 20 min until 60 min after increase to maximum dose	**Results:** *(Estimated from Fig. 1)* ***Assay Ab-1*** Range, pg/mL (μU/mL) 13–25 pg/mL (8–15 μU/mL) basal (in labour) 12–28 (7–17) with SOT infusion ***Assay Ab-2*** Mean pg/mL (μU/mL) < 0.8 pg/mL (< 0.5) basal (in labour) 3.0 (1.8) at 1 mU/min 4.7 (2.8) at 2 mU/min 6.7 (4.0) at 3 mU/min 8.4 (5.0) at 4 mU/min **Important findings:** Assay Ab-1: No significant rise with infusion Assay Ab-2: Linear dose-dependent rise (*p* < 0.01)	**Assay:** Analysed by 2 different RIA assays: Pitt Ab-1 antiserum Pitt Ab-2 antiserum **Comments:** Low-dose SOT regimen No rise with Ab-1 assay due to high basal levels vs. clear rise seen with Ab-2 assay Doubling infusion rate approx doubled oxytocin levels (Ab-2 assay) **Other reported data:** ***Basal oxytocin in 16 pregnant women at > 36wks*** (pg/mL ± SE (μU/mL)) Assay Ab-1: 12.9 ± 1.5 (7.7 ± 0.9) Assay Ab-2: 1.5.0 ± 0.3 (0.9 ± 0.2)
**Fuchs et. al. (1983)** Oxytocin and the initiation of human parturition. III. Plasma concentrations of oxytocin and 13,14-dihydro-15-keto-prostaglandin F2α in spontaneous and oxytocin-induced labor at term	**15 women** Labour induction with SOT **17 women** Spontaneous labour, no SOT infusion	**Dose regimen:** IV infusion 1-2 mU/min increased every 15 min **Maximum dose:** Range 1–16 mU/min **Sampling regimen**: Baseline and just before each increase in infusion rate	**Results:** Mean ± SEM, pg/mL ***With SOT infusion*** 17.4 ± 4.8 pg/mL pre-induction 21.1 ± 6.4 at 1–3 mU/min 49.1 ± 10.9 at 4–6 mU/min 58.8 ± 9.9 at 7–9 mU/min 110 ± 22.7 at 10–16 mU/min **Important findings:** Linear dose-dependent rise (p < 0.001) No SOT 15.4 ± 8.7 late pregnancy 19.9 ± 3.1 at < 2 cm 47.2 ± 7.6 at < 4 cm 46.3 ± 7.8 at 4-6 cm 43.0 ± 6.6 at 7-9 cm 45.8 ± 13.6 at 10 cm 45.4 ± 3.9 mean 1st stage	**Assay:** RIA **Comments:** Doubling infusion rate approx doubled oxytocin levels Oxytocin levels below 10 mU/min are within range of levels in women with no SOT
**Furuya et. al. (1988)** [Fundamental studies on the measurement of plasma concentration of oxytocin during perinatal period]	**5 women** Labour induction with SOT	**Dose regimen**: IV infusion 12 mU/min (20 ng/min) increased up to maximum 32 mU/min (53 ng/min) **Maximum dose:** Range 24-32 mU/min (40–53 ng/min) **Sampling regimen:** Baseline and with each increase	**Results:** *(Estimated from Fig. 7)* Mean ± variation, pg/mL Infusion rate mU/min (ng/min) ***Basal (pre-labour)*** 2 pg/mL ***With SOT infusion*** 8 ± 5 at 12 mU/min (20 ng/min) 17 ± 5 at 16 mU/min (26) 19 ± 8 at 20 mU/min (33) 38 ± 14 at 24 mU/min (40) 55 ± 6 at 32 mU/min (53) **Important findings:** Fig. 7 shows approx. Linear dose-response curve, although no statistics provided	**Assay:** RIA **Comments:** No control group Dose intervals not stated Variation statistic not stated Doubling infusion rate approx tripled oxytocin levels Data at highest infusion rate based on 3 samples **Other reported data:** ***Oxytocin with breastfeeding*** *(n = 1, estimated from Fig. 8)* 2.5 basal 14.3 peak at 20 min Peak levels similar to SOT infusion at 16 mU/min
**Perry et. al. (1996)** The pharmacokinetics of oxytocin as they apply to labor induction	**10 women** Labour induction with SOT	**Dose regimen:** 6 mU/min, increasing by 6 mU/min every 40 min to max 42 mU/min **Maximum dose**: Mean maximum dose: 22.8 ± 3.6 mU/min **Sampling regimen:** Baseline (in labour) and at intrauterine pressures of; 100–150, 150–200 and > 200 Montevideo units All samples taken > 40 min after change of SOT infusion rate	**Results:** Mean ± SEM, pg/mL ***Basal (pre-labour)*** 21.2 ± 0.9 pg/mL ***With SOT infusion*** 26.4 ± 1.3 at maximum uterine pressure **Important findings:** Oxytocin levels correlated with SOT infusion rate (*p* < 0.001) but not uterine pressure. (Data not provided)	**Assay:** RIA **Comments:** Small increment in oxytocin levels with quite high SOT infusion rates suggests insensitive assay **Other reported data:** ***Oxytocin MCR*** MCR independent of oxytocin levels (*p* = 0.71)
**Seitchik et. al. (1984)** Oxytocin augmentation of dysfunctional labor. IV. Oxytocin pharmacokinetics	See Amico et al. (1984)	See Amico et al. (1984)	See Amico et al. (1984)	Data from this study also published in Amico et al. (1984)
**Thornton et. al. (1990)** Effect of human pregnancy on metabolic clearance rate of oxytocin	**10 women** Labour induction with SOT **10 women** Administered SOT at 8–10 weeks postpartum (see below)	**Dose regimen** *(lLabour):* IV infusion 10.7 mU/min (17.9ng/min) increased after 30 min to 21 mU/min (35.7 ng/min) **Maximum dose:** 21 mU/min (35.1 mg/min) ( Postpartum**:** see below)- italics please **Sampling regimen:** 6 samples over 60 sec at baseline and 30 min, then 30 min after each infusion rate increase	**Results:** Mean ± SEM, pg/mL Infusion rate mU/min (ng/min) ***With SOT infusion*** 1.5 ± 0.3 pg/mL basal (pre-labour) 5.0 ± 0.5 at 10.7 mU/min (17.9 ng/min) 8.0 ± 0.9 at 21 mU/min (35.7) **Important findings:** These oxytocin levels were similar to those in postpartum women who were administered significantly lower SOT doses (see below), suggesting greater metabolism of SOT in pregnancy	**Assay:** RIA **Comments:** Doubling infusion rate approx doubled oxytocin levels **Other reported data:** ***Oxytocin MCR*** MCR independent of concentration and higher in pregnancy vs postpartum (*p* < 0.001). ***Plasma oxytocinase activity*** High oxytocinase activity in pregnancy (2.1 ± 0.02 IU/mL), very low postpartum (< 0.1 IU/mL)
**Intravenous infusion for labour induction or augmentation: sporadic measurements**				
**Arai T. (1980)** The significance of plasma oxytocin in pregnancy and at parturition Publication in Japanese Data obtained from full article translated into English	**14 women** Labour induction with SOT **16 women** Spontaneous labour, no SOT infusion or other interventions	**Dose regimen:** IV infusion Stated as 15 μU/min (=0.015 mU/min, extremely low dose More likely 15 mU/min) **Total dose, duration:** Not stated **Sampling regimen:** Before labour During 2nd stage labour	**Results:** Mean ± SD, pg/mL (μU/mL) ***Basal (40 wks gestation)*** 69.3 ± 17.7 (41.6 ± 10.6) (n = 8) ***With SOT infusion*** 120.1 ± 42.8 (71.9 ± 25.6) in labour ***No SOT*** 77.0 ± 28.2 (46.1 ± 16.9) in labour **Important findings:** Mean levels in labour significantly higher with SOT vs no SOT (*p* < 0.01)	**Assay:** RIA **Comments:** Oxytocin levels in labour with SOT approx double vs in labour without SOT **Other reported data:** ***Oxytocin patterns in a single contraction cycle*** Reported below ***Oxytocin levels following prostaglandin induction, pre-labour and in-labour caesarean, and caesarean following SOT treatment in labour*** (This data to be reviewed in subsequent systematic review^a^)
**De Tina et.al (2019)** Oxytocin and Oxytocinase in the Obese and Nonobese Parturients during Induction and Augmentation of Labor	**50 women** (25 obese & 25 non-obese) Labour induction with SOT	**Dose regimen:** IV infusion **Maximum dose:** 20 mU/min Total Dose: (mean ± SD) Nonobese women 3.4 ± 1.6 IU **Duration:** Nonobese women, 397 ± 159 min **Sampling regimen:** Baseline and 20 min after maximum infusion rate	**Results:** Non-obese women with complete data (*n* = 18) Median (IQR), pg/mL ***Basal (pre-labour)*** 351 pg/mL(56, 790) ***With SOT infusion*** 463 (15, 791) **Important findings:** No increase following SOT infusion	**Assay:** ELISA ELISA gives higher levels than RIA with different effect patterns **Comments:** Similar data in obese group, which was analysed separately in this study Low SOT dose (generally insufficient to increase plasma oxytocin) Small oxytocin rise may reflect labour-related release **Other reported data:** ***Oxytocin and oxytocinase levels in obese*** **vs.** ***non-obese women*** Oxytocin levels or total SOT dose not significantly different in obese vs non-obese women Lower initial oxytocinase levels in obese vs non-obese (p = 0.03)
**Husslein et. al. (1983b)** Oxytocin and the initiation of human parturition. IV. Plasma concentrations of oxytocin and 13,14-dihydro-15-keto-prostaglandin F2 alpha during induction of labor by artificial rupture of the membranes	**6 Women** Labour induction with ROM and later required SOT **16 women** Labour induction with ROM, no SOT	**Dose regimen:** IV infusion 5 mU/min Started 4–15 hr. after ROM when required **Maximum dose**: Not stated **Sampling regimen:** Before ROM, at 15 min and 2 hr., then 2–3 hourly	**Results:** Mean ± SEM, pg/mL ***With SOT infusion*** (after ROM) 38.3 ± 10.7 pg/mL basal (pre-induction) 59.9 ± 15.4 at 5 hr (3/6 with SOT) 53.0 ± 24.3 at 8 hr (2/3 with SOT) 63.1 ± 9.2 mean all samples ***No SOT*** 33.6 ± 7.7 basal (pre-induction) 40.6 ± 11 at 5 hr 32.9 ± 8.1 at 8 hr **Important findings:** Oxytocin levels significantly increased after vs before SOT infusion	**Assay:** RIA **Comments:** Oxytocin levels in labour with SOT approx double vs in labour without SOT **Other reported data:** ***Plasma PGFM (PG metabolite) following SOT infusion:*** PGFM increased in 6/6 women with successful induction ***Oxytocin levels following ROM*** (This data to be reviewed in subsequent systematic review^a^)
**Otsuki et al. (1983)** Feto-maternal plasma oxytocin levels in normal and anencephalic pregnancies.	**5 women** Labour induction with SOT **6 women** Spontaneous labour, no induction or SOT	**Dose regimen:** Not stated **Maximum dose:** Not stated **Sampling regimen:** Baseline, early labour and 2nd stage	**Results:** Mean ± SEM, pg/mL (reported in μU/mL) ***Basal (pre-induction)*** 20.7 ± 2.8 pg/mL (12.4 ± 1.7 μU/mL) ***With SOT infusion*** 40.7 ± 8.7 (24.4 ± 5.2) early labour 63.1 ± 12.9 (37.8 ± 7.7) 2nd stage ***No SOT*** 21.0 ± 3.0 (12.6 ± 1.8) early labour 29.4 ± 6.0 (17.6 ± 3.6) 2nd stage **Important findings:** SOT significantly higher vs no SOT at 1st (*p* < 0.05) and 2nd (*p* < 0.01) stages	**Assay:** RIA **Comments:** Oxytocin levels in labour with SOT approx double vs. in labour without SOT **Other reported data:** ***Maternal oxytocin levels in 4 women with anencephalic babies induced with SOT at 32–34 wk*** 22.4 ± 13.4 pg/mL 2nd stage Other reported data: ***Newborn UA and UV oxytocin levels with maternal SOT exposure*** (See Table 4)
**Padayachi et. al. (1988)** Serial oxytocin levels in amniotic fluid and maternal plasma during normal and induced labour	**8 women** Labour induction with SOT and amniotomy (women allocated alternately with PG induction) **13 women** Spontaneous labour, no SOT	**Dose regimen:** IV infusion at 2 mU/min increased every 30 min **Maximum dose:** 32 mU/min **Sampling regimen:** Collected over 4 min every hr.	**Results:** Mean ± SEM, pg/mL (μU/mL) ***With SOT infusion*** 5.9 ± 1.0 pg/mL at < 4 cm (3.55 ± 0.62 μU/mL) 10.1 ± 1.3 (6.07 ± 0.78) at 8–10 cm ***No SOT*** 6.8 ± 0.65 (4.05 ± 0.39) at < 4 cm 9.2 ± 1.15 (5.48 ± 0.69) at 8–10 cm **Important findings:** Significant rise during labour with SOT (*p* < 0.02)	**Assay:** RIA **Comments:** RIA giving very low levels in all groups Slow sampling may average out any peaks of oxytocin
**Pochard & Lutz-Bucher (1986)** Vasopressin and oxytocin levels in human neonates. Relationships with the evolution of labour and beta-endorphins	**6 women** Labour induction with ‘oxytocic drugs’ **21 women** No oxytocic drugs **10 women** No oxytocic drugs Fetal distress in labour	**Dose regimen:** Not stated **Maximum dose:** Not stated **Sampling regimen:** Before expulsive phase	**Results:** *(Estimated from Fig. 1)* Mean, pg/mL ***Oxytocic drugs*** 3 pg/mL ***No oxytocic drugs*** 2–3 ***Fetal distress*** 4 **Important findings:** No statistically significant differences between any groups.	**Assay:** RIA **Comments:** Oxytocic drugs presumed to be SOT **Other reported data:** ***Newborn UA and UV oxytocin with maternal SOT exposure*** (See Table 4) ***Maternal plasma and newborn UA and UV AVP (vasopressin) levels*** No difference between any groups ***Maternal plasma and newborn UA and UV oxytocin levels with pre-labour caesarean*** (This data to be reviewed in subsequent systematic review^a^)
**Risberg et. al. (2015)** Water balance during parturition and early puerperium: A prospective open trial	**12 women** Spontaneous labour, augmentation with SOT for ‘dystocia’(9/12 epidural, 5/12 postpartum SOT) **30 women** Spontaneous labour No IV fluids or other medication (14/30 postpartum SOT) **9 women** Spontaneous labour IV fluids only (7/9 epidural, 6/9 postpartum SOT) Women who required SOT were randomised to continuous (*n* = 6) or pulsatile (*n* = 5) infusions	**Dose regimen:** 10 IU/L (10 mU/mL) by continuous (n = 6) or pulsatile (n = 5) infusion: **Continuous infusion** 15 mL/hr. (2.5 mU/min) increased until adequate contractions (3–5/10 min) **Duration:** 72–480 min **Maximum dose:** 120 mL/hr. (20 mU/min) **Total dose:** 2.5 ± 1.1 IU total **Pulsatile infusion** 1.8 mL/hr. (0.3 mU/min) increased until adequate contractions **Duration:** 20–240 min **Total dose:** 0.4 ± 0.5 IU **Postpartum dose:** 25 of these women also received 10–30 IU SOT bolus **Sampling regimen:** Arrival, early 1st stage, early 2nd stage, just after birth and 9, 15, 27 hr. postpartum	**Results:** Mean ± SD, pmol/L equivalent to pg/mL ***With SOT infusion*** (Includes continuous and pulsatile infusion regimens) 31.3 ± 3.0 at arrival 29.4 ± 3.8 early 1st stage 29.5 ± 2.8 early 2nd stage 32.7 ± 3.3 just after birth 26.3 ± 2.5 9 hr. postpartum 28.8 ± 2.4 15 hr. postpartum 27.3 ± 3.6 27 hr. postpartum ***No SOT or IV fluids*** 32.4 ± 3.8 at arrival 20.7 ± 2.7 early 1st stage 29.3 ± 2.9 early 2nd stage 41.5 ± 8.3 just after birth 26.7 ± 1.2 9 hr. postpartum 28.0 ± 1.1 15 hr. postpartum 27.5 ± 1.3 27 hr. postpartum **Important findings:** Oxytocin concentrations not significantly different with vs without SOT or before vs after parturition	**Assay:** ELISA with previous extraction ELISA gives higher levels than RIA with different effect patterns **Comments:** Very low SOT dosage, especially with pulsatile dosage Multiple sub-groups and co-interventions make interpretation difficult High mean oxytocin just after birth in No SOT group may relate to mother-newborn SSC initiated soon after birth **Other reported data:** ***Pulsatile*** **vs** ***continuous SOT infusion*** Significantly lower total SOT dose (*p* < 0.05), no difference in labour progress ***SOT*** **vs.** ***controls*** SOT-exposed had lower plasma osmolarity at arrival (*p* < 0.05) and lower sodium (*p* < 0.01) in late labour ***AVP*** **vs.** ***controls*** AVP levels not significantly different between groups
**Seitchik et. al. (1985)** Oxytocin augmentation of dysfunctional labor. V. An alternative oxytocin regimen	**10 women** Labour augmentation with SOT	**Dose regimen:** IV infusion 5 mU/min increased by timed or arithmetic protocol until adequate uterine response **Maximum dose:** Range 2.3–5.7 mU/min **Sampling regimen:** Baseline (2 samples 5 min apart), at adequate uterine response, and every 15 min (up to 4 samples) after maintenance dose reached	**Results:** Mean ± SD, pg/mL (μU/mL) ***Basal (in labour)*** 1.4 ± 0.20 pg/mL (0.83 ± 0.1 μU/mL) ***With SOT infusion*** 2.6 ± 0.3 (1.6 ± 0.2) at adequate uterine response 3.0 ± 0.5 (1.8 ± 0.3) at 45 mins 2.8 ± 0.6 (1.7 ± 0.3) at 60 mins **Important findings:** Oxytocin levels approx doubled from basal at the infusion rate that gave adequate uterine response	**Assay:** RIA **Comments:** Oxytocin levels not analysed in relation to SOT infusion rate **Other reported data:** ***Time required to achieve steady-state blood oxytocin levels*** 40 min after dose increase
**Thornton et. al. (1992)** Plasma oxytocin during the first and second stages of spontaneous human labour	**5 women** Spontaneous labour, augmentation with SOT **7 women** Spontaneous labour, no SOT	**Dose regimen:** 4 mU/min increased every 15 min until adequate uterine activity **Total dose**: Not stated **Sampling regimen:** Every minute for 15 min in early labour (baseline) and in late 1st stage From onset 2nd stage pushing until birth	**Results:** Mean ± SD, pmol/L equivalent to pg/mL U: undetectable ***With SOT infusion*** U-4.6 pg/mL early labour U-10.0 late 1st stage ***No SOT*** U-6.2 early labour U-4.8 late 1st stage **Important findings:** Significant oxytocin rise following SOT infusion No significant oxytocin rise in 6/8 controls “Large increase” in oxytocin in 2nd stage in 2/8 women e.g. 27 pmol/L	**Assay:** RIA Assay detection limit 1.4 pmol/L **Comments:** Many samples with undetectable levels No increase during labour despite frequent sampling suggests insensitive assay Oxytocin levels in labour with SOT approx double vs in labour without SOT
**Intravenous infusion for labour induction or augmentation: oxytocin patterns during a single contraction cycle**				
**Arai T. (1980)** The significance of plasma oxytocin in pregnancy and at parturition Publication in Japanese Data obtained from full article translated into English	**14 women** Labour induction with SOT **16 women** Spontaneous labour, no SOT infusion or other interventions	**Dose regimen:** IV infusion Stated as 15 μU/min (=0.015 mU/min, extremely low dose More likely 15 mU/min) **Total dose, duration:** Not stated **Sampling regimen:** Sampled 4 times during a single contraction cycle in both 1st and 2nd stages	**Results:** Mean ± SD, pg/mL (μU/mL) ***Basal (40 weeks gestation)*** 69.3 ± 17.7 pg/mL (32.9 ± 10.6 μU/mL) ***Oxytocin levels in a single contraction cycle*** Sampled between, early, at peak and late in contractions: ***With SOT infusion:*** Stage 1 **(2 samples)** 107.9 ± 25.9 (64.6 ± 15.5) between contractions 98.2 ± 33.7 (58.5 ± 20.2) peak Stage 2: 97.2 ± 44.8 (58.2 ± 26.8) between 74.6 ± 1.2 (44.7 ± 0.7) early 102.2 ± 37.7 (61.2 ± 22.6) peak 64.1 ± 8.7 (38.4 ± 5.2) late **Important findings:** Levels significantly lower between vs. during contraction (Stage 1) ***No SOT:*** Stage 1 83.5 ± 17.5 (50.0 ± 10.5) between 70.0 ± 31.9 (41.9 ± 19.1) early 42.9 ± 15.4 (25.7 ± 9.2) peak 55.9 ± 28.4(33.5 ± 17.0) late Stage 2**:** 85.2 ± 16.4 (51.0 ± 9.8) between 70.0 ± 16.4 (41.9 ± 9.8) early 72.0 ± 29.1 (43.1 ± 17.4) peak 65.0 ± 30.7 (38.9 ± 18.4) late **Important findings:** Levels significantly lower at peak of contraction vs other times (Stage 1 only, *p* < 0.02)	**Assay:** RIA **Comments:** Differing patterns, with lowest oxytocin levels between contractions with SOT vs lowest at contraction peak with no SOT **Other reported data:** ***Oxytocin levels following prostaglandin induction, pre-labour and in-labour caesarean, and caesarean following SOT treatment in labour*** (This data to be reviewed in subsequent systematic review^a^)
**Buccal administration**				
**Dawood et. al. (1980)** Plasma oxytocin levels and disappearance rate after buccal Pitocin	**9 women** Labour induction or augmentation with SOT	**Dose regimen:** Buccal administration 400 IU every 20 min **Total dose:** 400–2200 IU **Sampling regimen:** Baseline, at 20–30 min, then hourly	**Results:** Range, pg/mL ***Basal (before SOT)*** 1.5–107 pg/mL ***With SOT buccal*** 6.8–181 peak **Important findings**: Very variable rise Levels not related to dose	**Assay:** RIA **Comments:** Buccal SOT is no longer used clinically **Other reported data:** ***Male subjects (3) given buccal SOT*** 200–400 IU every 20 min Mean levels 24–32 at 400 IU Still elevated above basal 45 min after administration
Postpartum intravenous or intramuscular administration				
**Ende et.al. (2019)** Association of Interindividual Variation in Plasma Oxytocin With Postcesarean Incisional Pain	**18 women** Pre-labour caesarean with postpartum IV SOT (all women)	**Dose regimen:** (Titrated to effect) *Intra-operative dose:* 3–35 IU (mean 14.1 ± 8.6 SD) *Post-operative dose:* 9–31.5 IU (mean 15.5 ± 4.8 SD) **Duration:** not stated **Total dose**: 19–48.5 (mean 29.5 ± 9.4 SD) **Sampling regimen:** I hr. before, then 1 and 24 hr post-caesarean	**Results:** Mean ± SD pg/mL (ng/mL) ***Mean (all women)*** 335 ± 452 pg/mL ***Range*** *(Estimated from Fig. A)* 1–1200 pre-caesarean (0.001–1.2 ng/mL) 30–1200 (0.03–1.2) 1 hr post-caesarean 4–1100 (0.004–1.1) 24 hr post-caesarean **Important findings:** Very high interindividual variability Significantly lower oxytocin at 1 and 24 hr. post- vs pre-caesarean (*p* = 0.001)	**Assay**: ELISA ELISA gives higher levels than RIA with different effect patterns **Comments:** Oxytocin data difficult to interpret Assay did not detect oxytocin rise at 1 hr. despite high intra- and post-operative SOT dose **Other reported data:** ***Pain score at 24 hr*** Significantly lower in women with higher oxytocin levels at 1 and 24 hr. post-caesarean (*p* = 0.047, *p* = 0.008 respectively)
**Fuchs et. al. (1982)** The origin of circulating 13,14-dihydro-15-keto-prostaglandin F2 alpha during delivery	**10 women** Postpartum SOT IV infusion **10 women** Postpartum IV infusion without SOT	**Dose regimen:** *In labour: *Some women also received SOT IV infusion 1–8 mU/min, stopped average 50 min before birth *Postpartum:* 100–150 mU/min starting immediately after birth for 2 hr., infusion rate reduced over time Total postpartum dose: 12–18 IU **Sampling regimen**: Full dilatation, then 5 min, 30 min and 2 hr. postpartum	**Results:** Mean ± SD, pg/mL ***Basal*** 18.0 ± 3.2 pg/mL pre-labour ***With SOT infusion*** 50.1 ± 9.7 2nd stage 93.4 ± 20.6 5 min postpartum 275 ± 46.5 30 min postpartum 127 ± 22.6 2 hr. postpartum ***No SOT*** 50.1 ± 10.7 2nd stage 41.1 ± 11.0 5 min postpartum 29.1 ± 9.7 30 min postpartum 29.4 ± 6.8 2 hr. postpartum **Important findings:** Postpartum oxytocin significantly higher with vs without SOT at 30 min and 2 hr. (both *p* < 0.001)	**Assay:** RIA **Comments:** Skin-to-skin contact not recorded **Other reported data:** ***PG levels postpartum*** Significantly higher PG levels at 2 hr. postpartum following postpartum SOT vs no postpartum SOT Data from this study also published in Husslein et al. (1982)
**Gibbens, D et.al** **(1972)** The circulating level of oxytocin following intravenous and intramuscular administration of Syntometrine	**10 W** SOT IM **14 W** SOT IV **2 W** SOT by subcutaneous injection (SC)	**Dose regimen:** Single injection at birth of baby’s anterior shoulder **Total dose**: 5 IU (combined with ergometrine 500 mcg) **Sampling regimen**: Basal then at varying intervals up to 120 mins: Frequent intervals 0–240 sec after IV (*n* = 4)	**Results:** *(Estimated from Fig. 1)* Mean, pg/mL **Basal:** Undetactable in all ***Intramuscular:*** 10 at 15 sec 20 at 30 sec 40 at 1 min 50 at 3 min 45 at 6 min 43 at 12 min 18 at 18 min 18 at 30 min 0 at 60 min ***Intravenous:*** 35 at 15 sec 330 at 30 sec 550 at 1 min 280 at 3 min 100 at 6 min 70 at 12 min 40 at 18 min 20 at 30 min 0 at 60 min **Important findings:** Following IM, much lower peak levels vs. IV, with a more sustained elevation	**Oxytocin assay:** RIA Max sensitivity reported as 1.5 pg/mL No oxytocinase inhibitor was added, suggesting that some degradation may have occurred **Comments:** Low basal levels suggests that actual peak levels may be higher than reported **Other reported data:** ***IM oxytocin levels*** Oxytocin first detected at 45 sec (average), still detectable at 60 min in 3/10 ***IV oxytocin levels*** Initial rapid fall (initial half-life 3 min) followed by slower disappearance IV levels at 20–220 sec (Fig. 2, n = 2) show peaks around 400–700 pg/mL at 40–80 sec with marked variability and decline to < 100 pg/mL at 220 sec ***SC oxytocin levels*** Levels undetectable (n = 1) and very low (39 pg/mL at 30 mins, *n* = 1)
**Husslein P et.al. (1983a)** [Oxytocin- and prostaglandin plasma concentrations before and after spontaneous labor: evidence of involvement of prostaglandins in the mechanism of placental separation]. Publication in German	See Fuchs et.al (1982)	See Fuchs et.al (1982)	See Fuchs et.al (1982)	Data from this study also published in Fuchs et.al (1982)
**Thornton et. al. (1988)** Plasma oxytocin during third stage of labour: comparison of natural and active management	**25 women** Postpartum SOT IM **15 women** No postpartum SOT	**Dose regimen:** Single IM injection of at birth of baby’s anterior shoulder **Total dose**: 5 IU (combined with ergometrine 500 mcg) **Sampling regimen:** Every 30 sec for 15 min from crowning of the head	**Results:** Mean ± SD, pmol/l equivalent to pg/mL With SOT IM postpartum 3.1 ± 2.0 pg/mL before anterior shoulder 15.9 ± 2.7 after birth Mean peak level 30.5 ± 2.5 No SOT In 9/15 women: 2.4 ± 3.1 before anterior shoulder 2.2 ± 2.2 after birth In 6/15 women: 3.2 ± 2.0 before anterior shoulder 6.4 ± 2.0 after birth Mean peak level 11.6 ± 1.5 **Important findings:** Significant rise following SOT (*p* < 0.001) In 6/15 with no SOT, significant rise (*p* < 0.01) with “remarkably similar” pattern to SOT	**Assay:** RIA
**Thornton et. al. (1990)** Effect of human pregnancy on metabolic clearance rate of oxytocin	**10 women** Administered SOT at 8–10 weeks postpartum (experimental) (SOT also administered in labour- see above)	**Dose regimen:** IV infusion 4.3 ng/min (2.6 mU/min) increased after 30 min to 8.5 ng/min (5.1 mU/min) **Maximum dose:** 8.5 ng/min (5.1 mU/min) **Sampling regimen:** 6 samples over 60 sec at baseline and 30 min, then 30 min after infusion rate increase	**Results:** Mean ± SEM Infusions, mU/min (ng/min) ***Basal*** 1.7 ± 0.5 pg/mL ***With SOT infusion*** 5.2 ± 0.4 pg/mL at 2.6 mU/min (4.3 ng/min) 8.0 ± 0.3 at 5.0 mU/min (8.5) **Important findings:** Oxytocin levels postpartum similar to labouring women who were administered significantly higher SOT doses (see above), suggesting greater metabolism of oxytocin in labour	**Assay:** RIA **Comments:** Doubling infusion rate approx doubled oxytocin levels, similar to dose-response elevations seen in labouring women in this study **Other reported data:** ***Oxytocin metabolic clearance rate (MCR)*** MCR independent of concentration and higher in pregnancy vs postpartum (*p* < 0.001) ***Plasma oxytocinase activity*** High in pregnancy (2.1 ± 0.02 IU/mL), very low postpartum (< 0.1 IU/mL)
**Velandia (2012)** Parent-Infant Skin-to-Skin Contact Studies	**34 women** Pre-labour caesarean Randomised to 25 min newborn SSC with mother or with father Control groups: the parent without SSC **All groups** Initial 5 min SSC with mother and were returned to mother after 30 min	**Dose regimen:** All women received 5 IU SOT IV after birth 19 women also received 50 IU SOT IV infusion over 90–158 min (316–555 mU/min) **Sampling regimen:** Basal (before surgery) At birth then every 5 min for 45 min, then every 15 min up to 120 min	**Results:** *(Estimated from Figs. 13, 14, 15)* Mean, pM equivalent to pg/mL ***SOT infusion & SSC (n = 7)*** 35 pg/mL basal 49 at 10 min 64 at 20 min 49 at 30 min 30 at 40 min 32 at 60 min 53 at 75 min ***No SOT infusion & SSC (n = 8)*** 22 basal 26 at 10 min 31 at 20 min 32 at 30 min 29 at 40 min 24 at 60 min 23 at 75 min ***SOT infusion, no SSC (n = 12***) 26 basal 37 at 10 min 37 at 20 min 32 at 30 min 33 at 40 min 31 at 60 min 50 at 75 min ***No SOT infusion, no SSC (n = 7)*** 29 basal 32 at 10 min 42 at 20 min 32 at 30 min 31 at 40 min 31 at 60 min 32 at 75 min **Important findings:** Rise from basal was significant at 20 and 75 min for women with SOT and SSC (*p* < 0.001) but not for other groups	**Assay:** RIA **Other reported data:** ***Oxytocin levels postpartum*** All groups of mothers and fathers with or without SSC had non-significant oxytocin rise over first 60 min, then levels decreased to basal ***SSC*** **vs** ***no SSC*** Fathers: no difference in oxytocin with SSC vs no SSC ***Newborns:*** Earlier first breastfeed when SSC with mother vs father (*p* = 0.018) Girls cried more in SSC with mother vs father (*p* = 0.004) ***Maternal personality profile at 2 days in relation to SOT and SSC*** Greatest personality changes on Karolinska Scale of Personality (vs. normative) in women with both SOT and SSC
**Yamaguchi et. al. (2011)** Serum oxytocin concentrations in elective caesarean delivery: A randomized comparison of three infusion regimens	**30 women** Pre-labour caesarean Randomised to 3 postpartum SOT IV regimens	**Dose regimen:** IV infusion started post-op (just after newborn cord clamped) Randomised to: **a.** 10 IU over 30 min (330 mU/min) **b**. 10 IU over 3 min (3330 mU/min) **c**. 80 IU over 30 min (2670 mU/min) **Total dose:** 10 or 80 IU **Sampling regimen:** Basal (pre-infusion) 5, 30 and 60 min after initiation of SOT infusion	**Results:** Mean ± SD pg/mL (reported in ng/mL) ***a. 10 IU over 30 min*** (330 mU/min) 60 ± 20 pg/mL (0.06 ± 0.02) basal 710 ± 270 (0.71 ± 0.270) at 5 min 1170 ± 370 (1.17 ± 0.37) at 30 min 650 ± 260 (0.65 ± 0.26) at 60 min ***b. 10 IU over 3 min*** (3330 mU/min) 40 ± 20 (0.04 ± 0.02) basal 1970 ± 650 (1.97 ± 0.65) at 5 min 410 ± 210 (0.41 ± 0.21) at 30 min 360 ± 260 (0.36 ± 0.26) at 60 min ***c. 80 IU over 30 min (2670 mU/min*** 70 ± 40 (0.07 ± 0.04) basal 3650 ± 740 (3.65 ± 0.74) at 5 min 6190 ± 1190(6.19 ± 1.19) at 30 min 80 ± 250 (0.08 ± 0.25) at 60 min **Important findings:** 8-fold higher dose (80 IU vs 10 IU) gives 5-fold higher levels at 5 and 30 min	**Assay:** ELISA with extraction ELISA gives higher levels than RIA with different effect patterns **Other reported data:** ***Uterine tone:*** Adequate in all groups, no additional uterotonic drugs required ***Cardiovascular:*** No significant changes in blood pressure or heart rate in any group, despite very high SOT dose
**Yuksel et. al. (2016)** Immediate breastfeeding and skin-to-skin contact during cesarean section decreases maternal oxidative stress, a prospective randomized case-controlled study	**90 women** Pre-labour caesarean Randomised to immediate vs. delayed (1 hour) newborn SSC	**Dose regimen:** 5 IU bolus after newborn cord clamped then 20 IU/hour infusion **Sampling regimen:** Basal (before surgery) 15 mins post-surgery	**Results:** Mean (range), pg/mL ***Delayed SSC*** 363.3 (187–645) pg/mL at 15 mins ***Immediate SSC*** 670.0 (435–890) at 15 mins **Important findings:** Oxytocin levels significantly higher with immediate vs delayed SSC (*p* = 0.003)	**Assay:** ELISA ELISA gives higher levels than RIA with different effect patterns **Comments:** All women received SOT **Other reported data:** ***Postoperative serum oxidative stress markers***: Oxidative stress markers more favourable with immediate vs delayed SSC (*p* < 0.001), and positively correlated with oxytocin levels (*p* < 0.001) ***SSC and pain markers:*** Women with immediate vs delayed SSC had non-significantly lower pain scores and lower analgesia requirements
**Administration of synthetic oxytocin with later postpartum sampling: exposed vs. unexposed analysis**				
**Handlin et. al. (2009)** Effects of sucking and skin-to-skin contact on maternal ACTH and cortisol levels during the second day postpartum-influence of epidural analgesia and oxytocin in the perinatal period	See Jonas (2009)	See Jonas (2009)	See Jonas (2009)	(Data from this study also published in Jonas et al. 2009)
**Jonas et. al. (2009)** Effects of intrapartum oxytocin administration and epidural analgesia on the concentration of plasma oxytocin and prolactin, in response to suckling during the second day postpartum	**Intrapartum:** **8 women** Labour augmentation with SOT **14 women** Labour augmentation with SOT plus epidural analgesia **20 women** Spontaneous labour, no SOT or other interventions **All women** primiparous	**Dose regimens:** *SOT IV infusion:* Total dose: Median 0.8 IU (1.35 μg) IQR 0.3–1.6 IU (0.53–2.73 μg) *SOT IV infusion with epidural* (SOT/EDA): Total dose: Median 0.9 IU (1.43 μg) IQR 0.4–1.8 IU (0.74–3.06 μg) **Sampling regimen:** During breastfeeding at 24–48 hr. postpartum with skin-to-skin contact (SSC) Baseline sample at first latch then 15 samples at 30 sec intervals over first 7.5 min and then 10, 20, 30, and 60 min	**Results:** A median of all samples for each woman was calculated, which were combined into group medians Median (IQR), pg/mL ***SOT/No EDA*** 156.3 pg/mL (102.0–376.2) basal 176.6 (161.2–193.3) at 1.5 min 171.3 (134.3–234.1) median all samples ***SOT/EDA*** 96.2 (82.6–123.0) baseline 121.4 (110.4–132.0) at 1.5 min 106.8 (96.7–157.1) median all samples ***No interventions*** 131.6 (97.1–227.2) basal 190.6 (121.0–234.6) at 1.5 min 144.1 (93.2–224.8) median all samples **Important findings:** Median all samples SOT/EDA significantly lower vs other groups (*p* = 0.033–0.005) with depressed profile, both basal and peak levels Higher SOT IV dose (in SOT IV and SOT/EDA groups) correlated with significantly lower median oxytocin (*p* = 0.019), mainly due to lower levels in SOT/EDA, see Fig. 3	**Assay:** ELISA ELISA gives higher levels than RIA with different effect patterns **Comments:** The combination of SOT and epidural gave rise to a unique depressed oxytocin effect pattern **Other reported data:** ***Oxytocin levels during breastfeeding at 2 days following labour epidural analgesia*** (This data to be reviewed in subsequent systematic review^a^) ***Blood pressure and personality changes in relation to SOT and other interventions*** (Data published elsewhere) Data from this study also published in Handlin et al. (2009)
	**Postpartum:** **13 women** Postpartum SOT IM, no SOT in labour **20 women** Spontaneous labour, no SOT or other interventions in labour or postpartum **All women** primiparous	**Dose regimens:** SOT postpartum IM injection: 10 IU (8.3 μg)^b^ **Sampling regimen:** During breastfeeding at 24–48 hr. postpartum with SSC Baseline at first latch then 15 samples at 30 sec intervals over first 7.5 min and at 10, 20, 30 and 60 min	**Results:** Median (interquartile range), pg/mL ***With SOT IM postpartum*** 159.3 pg/mL(100.1–219.6) basal 173.0 (122.1–205.8) at 1.5 min 158.2 (119–187.2) median all samples ***No SOT*** 131.6 (97.1–227.2) basal 190.6 (121.0–234.6) at 1.5 min 144.1 (93.2–224.8) median all samples **Important findings:** Median levels in SOT IM group not significantly different to No SOT group, with similar pattern of oxytocin release in response to breastfeeding	**Assay**: ELISA ELISA gives higher levels than RIA with different effect patterns **Other reported data:** ***Oxytocin levels during breastfeeding at 2 days following labour epidural analgesia*** (This data to be reviewed in subsequent systematic review^a^) ***Blood pressure and personality changes in relation to SOT and epidurals*** (Data published elsewhere)
**Takahashi et. al. (2021)** Epidural Analgesia With or Without Oxytocin, but Not Oxytocin Alone, Administered During Birth Disturbs Infant Pre-feeding and Sucking Behaviors and Maternal Oxytocin Levels in Connection With a Breastfeed Two Days Later [updated search 2022 Rev1]	**Intrapartum: 5 women** Labour augmentation with SOT **10 women** Labour augmentation with SOT plus EDA **13 women** Spontaneous labour, no SOT or other interventions **All women** primiparous	**Dose regimens:** SOT IV infusion: **Total doses:** Median 1.1 IU (1.86 μg) IQR 0.7–3.0 IU (1.145–5.046 μg) SOT IV infusion with epidural (SOT + EDA): Total dose: Median 2.5 IU (4.16 μg) IQR 0.89–3.78 IU (1.487–6.308 μg) **Sampling regimen:** (see Jonas above)	**Results:** Group means±SE, pg/mL *(Estimated from Fig. 2)* Mean: mean of OT levels from 0 to 60 min Variance: mean of OT variance between 0 and 7.5 mins ***SOT/No EDA*** 190 ± 80 mean 830 ± 200 variance ***SOT + EDA*** 110 ± 30 mean 820 ± 100 variance ***No interventions*** 160 ± 55 mean 780 ± 130 variance **Important findings:** No significant difference in mean OT levels or variance in SOT group vs controls. Lowest mean OT in SOT + EDA group (*p* < 0.005 vs SOT, p < 0.005 vs controls)	**Assay:** ELISA ELISA gives higher levels than RIA with different effect patterns **Comments:** Variance reflects pulsatility of oxytocin release **Other reported data:** ***Breastfeeding behaviour, IBFAT score:*** Shorter duration of rooting in: SOT vs controls (*p* < 0.05); SOT + EDA vs controls (*p* < 0.0001) and SOT vs SOT + EDA (*p* < 0.0001) ***Oxytocin levels during breastfeeding at 2 days following labour epidural analgesia*** (This data to be reviewed in subsequent systematic review^a^) ***Blood pressure, and personality changes in relation to SOT and other interventions*** (Data published elsewhere) Data from this study also published in Jonas et al. (2009) and Handlin et al. (2009)
	**Postpartum:** **8 women** Postpartum SOT IM (no SOT in labour) **13 women** Spontaneous labour, no SOT or other interventions in labour or postpartum **All women** primiparous	**Dose regimens:** SOT postpartum IM injection: 10 IU **Sampling regimen**: (see Jonas above)	**Results:** Group means±SE, pg/mL *(Estimated from Fig. 2)* Mean: mean of OT levels from 0 to 60 min Variance: mean of OT variance between 0 and 7.5 mins ***SOT IM postpartum*** 165 ± 50 mean 870 ± 200 variance ***No SOT (Controls)*** 160 ± 55 mean 780 ± 260 variance **Important findings:** No significant difference in mean OT levels or variance in SOT IM group vs. controls.	**Assay:** ELISA ELISA gives higher levels than RIA with different effect patterns **Comments:** Variance reflects pulsatility of oxytocin release **Other reported data:** ***Breastfeeding behaviour, IBFAT score***: Shorter duration of rooting in SOT IM vs controls (*p* < 0.0001) ***Oxytocin levels during breastfeeding at 2 days following labour epidural analgesia*** (This data to be reviewed in subsequent systematic review^a^) ***Blood pressure, cortisol levels and personality changes in relation to SOT and other interventions*** (Data published elsewhere) Data from this study also published in Jonas et al. (2009) and Handlin et al. (2009)
**Administration of synthetic oxytocin with later postpartum sampling: combined exposed/unexposed analysis**				
**Erickson et.al. (2020)** Oxytocin, Vasopressin and Prolactin in New Breastfeeding Mothers: Relationship to Clinical Characteristics and Infant Weight Loss	**46 women** Spontaneous labour With (11) and without (35) SOT augmentation in labour, birth 18/46 also received epidural analgesia 33/46 women also received postpartum SOT	**Dose regimen:** Not stated **Total or maximum dose:** ***Intrapartum*** Max infusion rate: 20 mU/min Max duration: 12 hours Total intrapartum dose: mean 1.3 IU (SD 1.3) ***Postpartum*** Dose not stated ***Total SOT*** (intra and postpartum) Mean 8.9 IU (SD 8.3) **Sampling regimen:** 4–5 days postpartum (*n* = 35) Blood sampled at breastfeeding commencement and 20 min later (*n* = 32 with > = 1 successful samples)	**Results:** (Oxytocin levels not given for women with SOT vs no SOT) Mean ± SD (all women) ***Basal*** 1641.5 ± 121.7 pg/mL baseline ***20 mins breastfeeding*** 1713.6 ± 127.2 **Important findings:** Significantly higher basal levels in women with higher SOT dose in labour (*p* = 0.03)	**Assay:** ELISA ELISA gives higher levels than RIA with different effect patterns **Comments:** Mixture of intra- and post-partum exposures Data used for correlations not provided **Other reported data:** ***Oxytocin in relation to birth and breastfeeding*** Significant increase in mean oxytocin (all women) at 20 min breastfeeding (*p* < 0.001) Higher basal oxytocin correlated with shorter labour (*p* = 0.003) ***AVP and prolactin during breastfeeding*** Women administered SOT in labour had increase in AVP levels from basal to 20 min breastfeeding, whereas women without SOT had reduced AVP (*p* = 0.03)
**Gu V. et.al. (2016)** Intrapartum Synthetic Oxytocin and Its Effects on Maternal Well-Being at 2 Months Postpartum Data includes several study populations, including two groups of women (stated as *n* = 287) who appear to be included in Prevost (2014)	**386 women** With and without SOT in labour, birth, postpartum 29/386 without SOT exposure included in combined analysis	**Dose regimen:** ***Intrapartum*** 2 mU/min increasing by 2 mU/min to maximum 20 mU/min ***Postpartum*** Intramuscular injection, stated as “standard dose 100 μg converted to 50 IU” **Total or maximum dose:** Mean 36.61 IU (SD 24.36) including postpartum dosage **Sampling regimen:** Single blood sample at home visit at 2 months postpartum (*n* = 318)	**Results:** (Oxytocin levels not given for women with SOT vs no SOT) ***Mean ± SD (all women)*** 281.02 ± 233.66 pg/mL **Important findings:** SOT total dose significantly predicted oxytocin levels at 2 months (*p* < 0.001) and accounted for 2.2% of the variance (data not provided) Women who were exclusively breastfeeding at 2 months postpartum had received significantly less SOT vs non-exclusively breastfeeding women (*p* < 0.001, data not provided)	**Assay:** ELISA, not extracted ELISA gives higher levels than RIA with different effect patterns **Comments**: Study design combines several populations Numbers of women included and sampled appear contradictory Reported intramuscular postpartum dose is very high (50 IU) in comparison to other included studies and to standard clinical practice (5-10 IU) Total dose is very high in comparison to other included studies **Other reported data:** ***SOT and mental health*** Higher SOT dose associated with greater (self-reported) depressive, anxious, and somatization symptoms (*p* < 0.05), and accounted for 4.7% of variance in depression symptoms
**Prevost et.al. (2014)** Oxytocin in pregnancy and the postpartum: relations to labor and its management. This study population appears to be also included in Gu (2016)	**272 women** Some women had SOT for induction or augmentation (numbers not stated)	**Dose regimen:** Not stated **Total or maximum dose**: *(Calculated from data in Table 2)* Total dose 0.58–3.02 IU Overall mean total dose: 1.4 IU **Sampling regimen:** Single blood sample at home visit at 2 months postpartum	**Results:** (Oxytocin levels not given for women with SOT vs no SOT) Mean ± SD, pg/mL ***All women*** 286.3 ± 272.7 **Important findings:** SOT total dose positively associated with oxytocin levels (data and significance not provided) Each extra IU SOT in labour increased oxytocin levels by 15.6 pg/mL (95% CIs: 5.7, 25.5)	**Assay:** ELISA, not extracted ELISA gives higher levels than RIA with different effect patterns **Comments:** Very high individual variability Study design combines two different populations Low doses unlikely to significantly raise oxytocin levels in labour No tests of significance given **Other reported data:** ***Oxytocin variability:*** Oxytocin (all samples, including during pregnancy) varied 70-fold between individuals (32.3–2297.6 pg/mL) ***Oxytocin and breastfeeding:*** Higher oxytocin levels (basal) in non-breastfeeding vs. breastfeeding women ***Oxytocin in pregnancy:*** Levels fell from early to late pregnancy in 73 women (27%)

Units: *IU* international units, *mU* milliUnits, *μU* microunits, *μg or mcg* micrograms, *ng* nanograms, *pg* picograms, *pmol* picomoles, *mL* millilitres

Oxytocin conversions: 1 IU = 1000 mU; 1 mU = 1000 μU; 1 mg = 1000 μg; 1 μg = 1000 ng; 1 ng = 1000 pg; 1 IU = 1.67 μg; 1 mU = 1.67 ng; 1 μU = 1.67 pg; 1 μg = 0.6 IU; 1 ng = 0.60 mU; 1 pg/mL = 1 pmol/L = 1pM

*Abbreviations*: *approx* approximately, *AVP* arginine vasopressin, *CI* confidence intervals, *cm* centimetres of cervical dilation, *EDA* epidural analgesia, *ELISA* enzyme-linked immunoassay (EIA also used), *fig* figure, *hr* hour, *IM* intramuscular, *IQR* interquartile range, *IV* intravenous, *MCR* metabolic clearance rate, *min* minutes, *PG* prostaglandin, *PGE2* prostaglandin E2, *PGF2α* prostaglandin F2alpha (13,14-dihydro-15-keto-prostaglandin F2 alpha), *PGFM* 13,14-dihydro-15-keto-PGF2α (PGF2alpha metabolite), *RIA* radioimmunoassay, *ROM* rupture of membranes, *SD* standard deviation, *SEM* standard error of the mean, *sec* seconds, *SOT* synthetic oxytocin, *SSC* skin-to-skin contact with newborn, *UA* umbilical artery, *UV* umbilical vein, *vs* versus, *wk* week

^a^Further systematic reviews (manuscripts in preparation) will report maternal and newborn plasma oxytocin levels in relation to caesarean section, epidural analgesia, opioid analgesia, prostaglandins, ROM and other interventions

^b^SOT postpartum intramuscular dose incorrectly reported in publication as 8.3 μg (10 IU), when actual dose was 16.7 μg =10 IU, as verified with authors

**Table 4:** Maternal synthetic oxytocin administration: newborn cord blood and maternal plasma oxytocin levels

Publication details	Number of participants Indication Dose regimen	Timing of maternal sampling	Maternal plasma (if sampled) and newborn cord umbilical artery (UA*) and umbilical vein (UV*) oxytocin levels, mean ± SEM, pg/mL (in original units)			Oxytocin assay Comments Important findings Other reported data
			Maternal	UA	UV	
**Maternal intrapartum synthetic oxytocin infusion**						
**Dawood et.al. (1978)** Fetal contribution to oxytocin in human labor	**7 women & newborns** Labour induction with SOT Dose regimen: Buccal and/or IV infusion Max. total dose 0.8-400 IU	No maternal samples	(Not sampled)	24.6 ± 9	69.9 ± 18.6	**Assay:** RIA **Comments:** Buccal SOT gives low maternal absorption **Important findings:** UA < UV oxytocin in SOT-exposed newborns (*p* < 0.01) (usually UA > UV, see studies below) **Other reported data:** Oxytocin levels in newborn UA and UV following pre-labour and in-labour caesarean (This data to be reviewed in subsequent systematic review^b^)
	**26 women & newborns** No SOT		(Not sampled)	116 ± 17.2	38 ± 5.6	
**Otsuki et. al. (1983)** Feto-maternal plasma oxytocin levels in normal and anencephalic pregnancies	**5 women & newborns** Labour induction with SOT Dose regimen: Not stated	During 2nd stage	63.1 ± 12.9 pg/mL (37.8 ± 7.7 μU/mL)	25.8 ± 4. (15.2 ± 2.5)	22.2 ± 5.5 (13.3 ± 3.3)	**Assay:** RIA **Important findings:** No significant differences in UA or UV oxytocin for newborns of SOT-exposed vs unexposed mothers **Other reported data:** Significantly higher oxytocin in women with vs without SOT (*p* < 0.001) No significant difference in 2nd stage oxytocin in women with anencephalic vs unaffected fetus (no SOT in either group) Oxytocin levels in newborn UA and UV following pre-labour caesarean (This data to be reviewed in subsequent systematic review^a^)
	**6 women & newborns** No SOT		29.4 ± 6.0 (17.6 ± 3.6)	30.4 ± 8.2 (18.2 ± 4.9)	20.4 ± 3.8 (12.2 ± 2.3)	
	**4 women & newborns** No SOT Anencephalic babies		22.4 ± 13.4 (13.4 ± 8.0)	(Not detectable)	(Not detectable)	
**Padayachi et. al. (1988)** Serial oxytocin levels in amniotic fluid and maternal plasma during normal and induced labour	**8 women & newborns** Labour induction with SOT Dose regimen: 2–32 mU/min	Collected over 4 min at 8–10 cm	10.1 ± 1.3 pg/mL (6.07 ± 0.8 μU/mL)	67 (40)	37 (21)	**Assay:** RIA **Comments:** *UA and UV oxytocin estimated from Fig. 3* All induced women also had ROM **Important findings:** No significant differences in UA or UV oxytocin for SOT-exposed vs unexposed newborns **Other reported data:** Oxytocin levels in amniotic fluid (AF): No significant difference in AF oxytocin in SOT-exposed vs unexposed in early or late labour Oxytocin levels in newborn UA and UV following prostaglandin-induced labour and pre-labour caesarean (This data to be reviewed in subsequent systematic review^b^)
	**13 women & newborns** No SOT		9.1 ± 1.2 (5.48 ± 0.6)	30 (18)	17 (10)	
**Patient et. al. (1999)** The effect of labour and maternal oxytocin infusion on fetal plasma oxytocin concentration	**10 women & newborns** SOT infusion **Dose regimen:** 2–28 mU/min, mean 10 mU/min	No maternal samples	(Not sampled)	17.7 pg/mL (95% CIs: 9.7–32.1)	7.3 (4.6–11.8)	**Assay:** RIA **Comments:** pmol/L equivalent to pg/mL **Important findings:** UA > UV all groups (*p* < 0.01) No significant differences in UA or UV oxytocin or UA:UV ratio for newborns of SOT-exposed vs unexposed mothers **Other reported data:** Oxytocin levels in newborn UA and UV following maternal pethidine in labour, pre-labour and in-labour caesarean (This data to be reviewed in subsequent systematic review^b^)
	**15 women & newborns** No SOT		(Not sampled)	14.8 (8.7–25.1)	7.8 (4.7–13.1)	
**Pochard & Lutz-Bucher (1986)** Vasopressin and oxytocin levels in human neonates. Relationships with the evolution of labour and beta-endorphins	**6 women & newborns** Labour induction or augmentation with ‘oxytocic drugs’ (presume SOT) Dose regimen: Not stated	At time of birth	3 pg/mL	36	13	**Assay:** RIA **Comments:** *All oxytocin levels estimated from Fig. 1* **Important findings:** UA oxytocin correlated with UV levels (*p* < 0.01) UA > UV levels (*p* < 0.0001) and UV > maternal levels (*p* < 0.0002) No significant difference in UA or UV oxytocin for newborns of SOT-exposed vs unexposed mothers or following fetal distress **Other reported data:** Oxytocin levels in newborn UA and UV following pre-labour caesarean (This data to be reviewed in subsequent systematic review^b^) Newborn AVP levels: UA > UV (*p* < 0.001) and UV > maternal (*p* < 0.0001) all groups
	**21 women & newborns** No SOT		2	18	8	
	**10 women & newborns** Following fetal distress in labour		4	22	7	
**Maternal postpartum synthetic oxytocin**						
**Sellers et. al. (1981a)** Is oxytocin involved in parturition?	**9 women & newborns** Postpartum SOT (No SOT in labour) **Dose regimen:** Combination of SOT 5 IU and ergometrine 500 μg Given IM with birth of anterior shoulder	No postpartum maternal samples	(Not sampled)	75	35	**Assay:** RIA **Comments:** *UA and UV levels estimated from Fig. 2* Timing of cord clamping not stated **Important findings:** No significant difference in UA or UV oxytocin for newborns of SOT-exposed vs unexposed mothers Oxytocin levels in UA > UV (*p* < 0.05) in controls **Other reported data:** Oxytocin levels in newborn UA and UV following pre-labour caesarean (This data to be reviewed in subsequent systematic review^b^)
	**8 women & newborns** No postpartum SOT/ergometrine		(Not sampled)	115	50	

Units: *IU* international units, *mU* milliUnits, *μU* microunits, *mg* milligrams, *μg or mcg* micrograms, *ng* nanograms, *pg* picograms, *pmol* picomols, *mL* millilitres

Oxytocin conversions: 1 IU = 1000 mU; 1 mU = 1000 μU; 1 mg = 1000 μg; 1 μg = 1000 ng; 1 ng = 1000 pg; 1 IU = 1.67 μg; 1 mU = 1.67 ng; 1 μU = 1.67 pg; 1 μg = 0.6 IU; 1 ng = 0.60 mU; 1 pg/mL = 1 pmol/L = 1pM

*Abbreviations*: *AF* amniotic fluid, *approx* approximately, *AVP* arginine vasopressin, *CI* confidence intervals, *cm*. centimetres of cervical dilatation, *fig*. Figure, *IM* intramuscular, *IV* intravenous, *MCR* metabolic clearance rate, *min* minutes, *RIA* radioimmunoassay, *ROM* rupture of membranes, *SE* standard error, *SEM* standard error of the mean, *SOT* synthetic oxytocin, *UA* umbilical artery, *UV* umbilical vein, *vs* versus

^a^UA and UV: UA carries blood from fetus to placenta, UV carries blood from placenta back to fetus

^b^Further systematic reviews (manuscripts in preparation) will report maternal and newborn plasma oxytocin levels in relation to caesarean section, epidural analgesia, opioid analgesics and other interventions

Results are also presented in text below, and both tables and text are grouped according to patients sampled (women, newborn); time and method of synthetic oxytocin administration (before labour, in labour, postpartum); and timing and frequency of plasma sampling.

In addition, Table 5: ‘Synthetic oxytocin dose regimens, maternal levels and total dose calculations’ presents a summary of several relevant studies to allow comparisons of dose-response data.

**Table 5:** Synthetic oxytocin dose regimens, maternal levels and total dose calculations

Synthetic oxytocin (SOT): Infusion rate (mU/min) OR Intramuscular injection in International Units (IU) OR Controls (Sampling details)	Maternal oxytocin levels (mean, pg/mL)	Calculated total SOT (IU) per hour	Calculated total SOT (IU) if dose given over 8 hours (unless stated)	Comments
**Intrapartum**				
***Data from Fuchs (1983) n = 15 labour induction, n = 17 spontaneous labour***				
Basal levels pre-labour	17.4	Nil	Nil	Doubling infusion rate approx doubled oxytocin levels Oxytocin levels below 10 mU/min are within range of levels in women with no SOT (See Table 3 for details)
Spontaneous 1st stage	45.4	Nil	Nil	
SOT: 1–3 mU/min	21.1	0.06–0.18	0.5–1.4	
4–6 mU/min	49.1	0.24–0.36	1.9–2.9	
7–9 mU/min	58.8	0.42–0.54	3.4–4.3	
10–16 mU/min	110	0.6–0.96	4.8–7.7	
***Data from Furuya (1988) n = 5 labour induction (Data estimated from Fig. 7)***				
Basal levels pre-labour	2			No control group without SOT Doubling infusion rate approx tripled oxytocin levels
SOT: 12 mU/min	8	0.6 IU	4.8	
16 mU/min	17	0.9	7.2	
20 mU/min	19	1.2	9.6	
24 mU/min	38	1.4	11.2	
32 mU/min (n = 3)	55	1.9	15.2	
**Postpartum (vaginal birth)**				
***Data from Thornton (1988) n = 25 postpartum SOT, n = 15 no postpartum SOT*** ***Sampled every 30 sec for 15 min, starting just before birth***				
No SOT:		Nil	Nil	Standard postpartum intramuscular injection (5 IU SOT with 0.5 mg ergometrine) Peak oxytocin levels 3-fold higher than peak physiological levels, as measured in this postpartum study Total dose: 5 IU SOT
before birth	3.2			
at 5–15 min	6.4			
mean peak	11.6			
5 IU intramuscular (IM) SOT at birth of anterior shoulder:		5	5 (single dose)	
before birth	3.1			
at 5–15 min	15.9			
mean peak	30.5			
***Data from Gibbens (1972) n = 10 postpartum SOT IM; n = 14 postpartum SOT IV bolus*** ***Sampled frequently for 60 min, starting just before birth (Data estimated from Fig. 1)***				
SOT IM:		Nil	Nil	Standard postpartum injection (5 IU SOT with 0.5 mg ergometrine) given IM/IV at birth of anterior shoulder Total dose: 5 IU SOT Rapid high peak following IV bolus, peaking at 1 min Gradual lowering but prolonged peak following IM
before birth	< 1.2			
at 1 min	40			
at 3 min	50			
at 6 min	45			
at 30 mins	18			
SOT IV:		5	5 IU (single dose)	
before birth	< 1.5			
at 30 sec	330			
at 1 min	550			
at 3 min	280			
at 6 min	100			
at 30 min	20			
***Data from Fuchs (1982) n = 10 postpartum SOT, n = 10 no postpartum SOT*** ***Sampled just before birth and 5 min, 30 min, 2 hours postpartum***				
No SOT:		Nil	Nil	Postpartum infusion dose relatively high compared to labour Oxytocin levels 8–10-fold higher than physiological levels, as measured in this postpartum study Total dose: 12-18 IU SOT
at 5 min	41.1			
at 30 min	29.1			
at 120 min	29.4			
SOT infusion at 100–150 mU/min, reduced over 2 hours		6–9	12–18 (over 2 hours)	
at 5 min	93.4			
at 30 min	275			
at 120 min	127			

Units: IU: international units; mU: milliUnits; pg: picograms; mL: millilitres

Oxytocin conversions: 1 IU = 1000 mU; 1 mU = 1000 μU; 1 mg = 1000 μg; 1 μg = 1000 ng; 1 ng = 1000 pg; 1 IU = 1.67 μg; 1 mU = 1.67 ng; 1 μg = 0.6 IU; 1 ng = 0.60 mU; 1 pg/mL = 1 pmol/L = 1pM

*Abbreviations*: *approx* approximately, *IM* intramuscular, *IV* intravenous, *min* minutes, *sec* seconds, *SOT* synthetic oxytocin

See Table 3 for full details of studies

### Effect of administration of synthetic oxytocin on maternal plasma oxytocin levels

#### Administration of synthetic oxytocin before labour

Fuchs and colleagues (1991) administered a single intravenous bolus of synthetic oxytocin to 18 pregnant women at term at doses of 2, 4, 8 or 16 milliunits (mU) [5]. Boluses of 4 mU or higher caused significant, peak-shaped elevations of maternal plasma oxytocin at 1–2 minutes after administration. Oxytocin peak levels were similar to levels during spontaneous pulses in labour, as measured in this study. This bolus administration of synthetic oxytocin gave rise to uterine contractions in most women. At each dose level (2, 4, 8 or 16 mU) the average number of contractions during the first 10 minutes was correlated with mean peak maternal oxytocin levels.

#### Administration of synthetic oxytocin to induce or augment labour

##### Intravenous infusion of synthetic oxytocin

**Dose-response data**

Six studies (reported in seven publications) reported maternal plasma oxytocin levels in relation to differing, specified infusion rates of synthetic oxytocin to induce or augment labour [42, 43, 50, 51, 61, 65, 70]. Dose-response relationships between the rate of infusion of synthetic oxytocin and maternal plasma oxytocin levels in these studies are reported in Table 3.

Infusion rates in labour varied from 1 to 2 to 32 milliunits per minute (mU/min). In one study, the theoretical maximum dose was 42 mU/min but the actual mean maximum was 22.8 mU/min [61]. A linear relationship was found between the infusion rate and maternal plasma oxytocin levels in all studies. Doubling the infusion rate caused an approximate doubling of oxytocin levels, clearly seen in Fuchs (1983) [50]. Doubling the infusion rate approximately tripled levels in Furuya (1988), although numbers were small and data less clear in this Japanese-language study [51].

Several of these dose-response studies also measured plasma oxytocin levels in women without synthetic oxytocin infusions, allowing a comparison between oxytocin levels during normal (physiological) birth and with infusions of synthetic oxytocin. For example, Fuchs (1983) measured oxytocin levels during spontaneous labour and birth and found that oxytocin levels rose progressively, increasing from around 20 pg/mL at less than 2 cm cervical dilation (cm) to 46 pg/mL at 10 cm (full dilation). In the same study, synthetic oxytocin infusions at 7–9 mU/min increased oxytocin levels from pre-induction levels (around 17 pg/mL) to 59 pg/mL, within the range of levels in late spontaneous labour. At the higher infusion rate of 10–16 mU/min, maternal plasma oxytocin levels were doubled to 110 pg/mL, which is around twice maximum oxytocin levels in women in late spontaneous labour in this study [50].

**Comments: dose-response data**

These studies contribute significantly to this review as they are very well structured, include many samples and use reliable assays.

These studies also valuably illustrate the influence of assay sensitivity on oxytocin levels. In assays that give very low basal levels, for example, Amico (1984)/Seitchik (1984) and assay Ab-1 in Amico (1986), the rise of oxytocin levels was observable in response to low infusion rates (1–4 mU/min) and gave a consistent dose-response relationship [42, 43, 65]. In contrast, assays that gave higher basal oxytocin levels such as Fuchs (1983) and assay Ab-2 in Amico (1986) tended to mask the small oxytocin increases at low infusion rates [43, 50]. However, elevations were seen at higher infusion rates in Fuchs (1983) [50]. See Table 3 for full details.

**Sporadic measurements without dose-response data**

Nine studies reported one or more maternal plasma oxytocin levels in labour in relation to different infusion rates of synthetic oxytocin (between 2 and 32 mU/min) but data in these studies was insufficient to allow dose-response calculations [20, 46, 56, 58, 59, 62, 64, 66, 71].

In five of these nine studies, women administered synthetic oxytocin in labour (doses not clearly reported) had plasma oxytocin levels that were approximately doubled compared to basal (pre-labour) levels or to levels in women without synthetic oxytocin [20, 56, 58, 66, 71].

In three studies, no elevation of plasma oxytocin was found in response to administration of synthetic oxytocin [46, 62, 64]. However, these studies also did not detect any oxytocin rise during labour in women without synthetic oxytocin, suggesting that the assay sensitivity was insufficient to detect the differences in oxytocin levels, and/or that the synthetic oxytocin dosage was very low. This may also apply to Padayachi (1988), who found a small oxytocin rise for women administered synthetic oxytocin but no rise during labour without synthetic oxytocin [59]. See Table 3 for full details.

**Comments: sporadic measurements**

These studies lack detail in relation to infusion rates of synthetic oxytocin and involve assays of differing sensitivity and quality. However, the data support the findings from dose-response curves that administration of synthetic oxytocin raises maternal plasma oxytocin levels only moderately: up to doubled levels in most studies.

**Oxytocin patterns during a single contraction cycle**

Using frequent sampling, Arai (1980) sampled maternal plasma oxytocin four times during single contractions in women labouring without interventions and women administered synthetic oxytocin [20]. Intrauterine pressure was simultaneously monitored.

In women without administration of synthetic oxytocin or other interventions, plasma oxytocin levels varied during contractions. Perhaps unexpectedly, maximal oxytocin levels were observed between contractions, with the lowest oxytocin levels coinciding with the contraction peak. However, in women administered infusions of oxytocin, this fluctuating pattern was not observed. Plasma levels were lower between contractions and the pattern was more flat, consistent with the constant infusion. See Table 3 for detailed data.

##### Buccal administration of synthetic oxytocin 

Dawood and colleagues (1980) studied the effects of buccal administration of synthetic oxytocin to induce or augment labour on maternal plasma oxytocin levels [45]. (Buccal administration involves synthetic oxytocin being placed adjacent to the buccal mucosa in the mouth, between the cheek and teeth.) Relatively high doses were used in this study: 400 IU every 20 minutes. Buccal absorption was very low and peak plasma levels were highly variable (6.8–181 pg/mL). Buccal administration has since been discontinued due to its low and unpredictable rate of absorption.

#### Administration of synthetic oxytocin postpartum

Nine studies (reported in twelve publications) reported maternal plasma oxytocin levels in response to postpartum synthetic oxytocin administration [47, 49, 52, 54, 55, 57, 68–70, 72–74].

Four of these studies reported maternal oxytocin levels during post-caesarean intravenous infusions at different rates and duration [47, 72–74]. Fuchs and colleagues (1982) reported similar data following vaginal birth, also published in Husslein (1983) [49, 55]. Thornton and colleagues (1988) reported maternal plasma levels following postpartum intramuscular injection of synthetic oxytocin, and Gibbens and colleagues (1972) reported maternal plasma oxytocin levels following synthetic oxytocin administered by intramuscular, intravenous or subcutaneous injection [52, 69]. Thornton (1990) administered synthetic oxytocin by intravenous infusion experimentally to women at eight to ten weeks postpartum [70].

One study, reported in three publications (Handlin (2009) and Jonas (2009) and more recently reanalysed by Takahashi and colleagues, (2021)) described plasma oxytocin levels during breastfeeding 2 days postpartum in relation to postpartum intramuscular injection of synthetic oxytocin and also in relation to intravenous infusion during labour [54, 57, 68]. This study, and the three publications, are discussed in detail below: ‘Administration of synthetic oxytocin with later postpartum sampling: exposed vs. unexposed analysis.’

##### Intravenous administration

Fuchs and colleagues (1982) measured plasma oxytocin levels in healthy women who were administered an infusion of synthetic oxytocin following vaginal birth at 100–150 mU/min, with the infusion rate decreasing over 2 hours (total dose 12–18 IU) [49]. Plasma oxytocin levels in women receiving this infusion were almost ten times higher at 30 minutes postpartum than corresponding levels in unmedicated women (275 vs. 29 pg/mL) and were still elevated at 2 hours (127 vs. 29 pg/mL), when infusion rates had declined.

Gibbens and colleagues (1972) administered synthetic oxytocin (5 IU, with ergometrine 0.5 mg) by intravenous injection to 14 women following vaginal birth and took frequent samples for up to 90 minutes [52]. Oxytocin levels rose rapidly from undetectable (less than 1.5 pg/mL) pre-injection to 550 pg/mL (mean) at 3 min, then fell rapidly. There was a more prolonged decrease after 6 minutes, and oxytocin was not detectable at 60 minutes in any woman. Initial half-life was calculated as 3 minutes from this data. Individual women’s oxytocin levels peaked as high as 700 pg/mL at 40–60 seconds in this study.

Yamaguchi and colleagues (2011) reported maternal plasma oxytocin levels following synthetic oxytocin infusions to healthy women after pre-labour caesarean who were randomised to three different regimes: 10 IU over 30 minutes (equivalent to 333 mU/min); 10 IU over 3 minutes (3333 mU/min); and 80 IU over 30 minutes (2666 mU/min) [73]. Plasma samples were taken at basal (pre-caesarean) and 5, 30 and 60 minutes after the infusion commenced in each group. Plasma oxytocin levels were analysed with ELISA, which gives higher levels than RIA. Maternal oxytocin rose rapidly in all groups, reaching approximately 50% of maximum levels by 5 minutes. Women who received 80 IU over 30 minutes (2670 mU/min, group c) had oxytocin levels that were 5-fold higher at 5 and 30 minutes than those administered 10 IU over the same period (330 mU/min, group a). When 10 IU was given more rapidly over 3 minutes (3330 mU/min, group b), plasma levels increased 50-fold from basal at 5 minutes. Plasma oxytocin levels were still around 10-fold elevated over basal at 60 minutes in all groups, including women administered 10 IU over 3 minutes. No haemodynamic side-effects were detected at any dose. See Table 3 for detailed data.

Velandia (2012) measured oxytocin levels after pre-labour caesarean in mothers and fathers randomised to 25 minutes of skin-to-skin contact (SSC) with their newborn babies or not, following 5 minutes of maternal SSC [72]. All women had received intravenous synthetic oxytocin 5 IU after birth, and half of the women received an additional 50 IU infusion over 1.5–2 hours postpartum. All groups showed small elevations in plasma oxytocin over the first hour, but this rise was only significant in those women with SSC who also received additional synthetic oxytocin. In these women, mean levels peaked at 64 pg/mL at 20 mins, compared to 35 pg/mL basal.

Yuksel and colleagues (2015) measured oxytocin levels following pre-labour caesarean in women randomised to SSC and breastfeeding with their newborns either immediately (in the operating room) or after 1 hour delay [74]. All women received synthetic oxytocin 5 IU by intravenous bolus followed by 20 IU/hour infusion. Maternal plasma was sampled for oxytocin levels (ELISA assay) before spinal anaesthesia was administered and again at 15 minutes post-caesarean. Women with immediate SSC had significantly higher oxytocin at 15 minutes (670.0 pg/mL) compared with women with delayed SSC (363.3 pg/mL) (*p* = 0.003). See Table 3 for full details.

In an experimental study, Thornton and colleagues (1990) administered low-dose synthetic oxytocin infusion to women at 8–10 weeks postpartum at 2.6 mU/min, increased to 5 mU/min after 30 minutes [70]. This approximate doubling of the infusion rate increased mean plasma oxytocin levels 1.5-fold (5.2 to 8.0 pg/mL).

##### Intramuscular injection

Thornton and colleagues (1988) sampled plasma oxytocin levels frequently (every 30 seconds for 15 minutes) in women with and without an intramuscular injection of synthetic oxytocin (5 IU in combination with ergometrine 0.5 mg, ‘Syntometrine’) at delivery of the baby’s anterior shoulder [69]. Oxytocin levels rose over several minutes from basal 3–16 pg/mL to a mean peak of 30 pg/mL. Among the women not administered synthetic oxytocin, six out of 15 had “remarkably similar” levels and patterns of oxytocin release, with a rise from 3.2 to 6.4 pg/mL and a mean peak of 11.6 pg/mL, whereas nine women (two-thirds) had no significant rise. The presence or absence of SSC was not reported.

Gibbens and colleagues (1972) also measured plasma oxytocin levels at frequent intervals following Syntometrine (5 IU synthetic oxytocin with 0.5 mg ergometrine) intramuscular injection [52]. Oxytocin was detectable in plasma from as early as 30 seconds, and average levels showed a gradual rise, with a wide peak (50 pg/mL) from 3 to 12 minutes. Oxytocin plasma levels remained elevated above basal (less than 1.5 pg/mL) for 30 minutes in all women, and as long as 60 mins in several women. There were no measurements of plasma oxytocin levels in untreated women in labour or postpartum for comparison in this study.

##### Comments: postpartum synthetic oxytocin

Postpartum intravenous infusions of synthetic oxytocin involved higher infusion rates, giving higher maternal plasma oxytocin levels, than during labour (see Tables 3 and 5). However, the duration of the postpartum infusion was generally shorter, so the total postpartum dose of synthetic oxytocin following vaginal birth (12–18 IU intravenous) was generally comparable to the intrapartum dose in the included studies. Frequent sampling following a 5 IU IV bolus after vaginal birth found very high but transient oxytocin elevations. Plasma oxytocin showed a slower and lower response to intramuscular injection of synthetic oxytocin (5 IU with 0.5 mg ergometrine) following vaginal birth [52, 69]. Total postpartum doses of synthetic oxytocin administered following caesarean (5–55 IU) tended to be higher than total postpartum doses following vaginal birth (5–18 IU).

#### Administration of synthetic oxytocin with later postpartum sampling: exposed vs. unexposed analysis

Jonas and colleagues (2009), also reported by Handlin and colleagues (2009), sampled oxytocin levels frequently during a breastfeeding episode two days after birth in primiparous women grouped according to medical interventions in labour and birth, including synthetic oxytocin administered by intravenous infusion during labour and by intramuscular injection (10 IU) postpartum [54, 57]. A subset of this data was reanalysed by Takahashi (2021) [68].

Jonas/Handlin report median total doses of intrapartum and intramuscular postpartum synthetic oxytocin as 0.9 IU and 10 IU respectively, and the Takahashi subset reported 1.1 IU intrapartum and 10 IU postpartum total doses [54, 57, 68]. Analysis was performed using ELISA, and data allowed analysis of dose-exposure relationships, as well as interactions between synthetic oxytocin and other interventions.

The combination of synthetic oxytocin with epidural analgesia was associated with significantly lower oxytocin levels throughout the breastfeeding episode, compared to women with neither exposure. There was also a dose-response pattern, with a higher amount of synthetic oxytocin infused in labour (with epidural co-intervention) associated with lower oxytocin release in women during breastfeeding. Women who received only a postpartum intramuscular injection of synthetic oxytocin, without synthetic oxytocin or other interventions in labour, had oxytocin levels and patterns during breastfeeding that were equivalent to women without any obstetric medications.

These findings were confirmed in a re-analysis by Takahaski and colleagues (2021), which found the lowest mean oxytocin levels during breastfeeding in women with epidural and synthetic oxytocin vs. synthetic oxytocin alone (*p* < 0.005) or controls (*p* < 0.005).

Similarly, this analysis found no significant difference in mean plasma oxytocin levels between women with and without a postpartum intramuscular injection (10 IU) of synthetic oxytocin (both groups had no intrapartum synthetic oxytocin infusions).

##### Comments: synthetic oxytocin with later postpartum sampling: exposed vs. unexposed analysis

The low intrapartum total doses in these study (0.8–1.1 IU) would not be expected to raise maternal plasma oxytocin significantly above physiological levels, according to data in this review. However, the data from women exposed to epidural analgesia in addition to synthetic oxytocin during labour suggest that this combination may have significant impacts on postpartum maternal oxytocin systems. (The effects of epidural analgesia on maternal and newborn plasma oxytocin levels will be reported in an upcoming systematic review.) It is of interest that postpartum intramuscular administration alone (10 IU) did not affect oxytocin release during breastfeeding on day two postpartum.

#### Administration of synthetic oxytocin with later postpartum sampling: combined exposed-unexposed analysis

At four to five days postpartum, Erickson and colleagues (2020) measured plasma oxytocin levels twice during a breastfeeding episode (basal and at 20 minutes) in 46 women: 11 had received synthetic oxytocin for labour augmentation, 33 received synthetic oxytocin postpartum (dose not stated) and 18 received epidural analgesia [48]. Analysis was performed using ELISA. Levels were not compared between exposed and unexposed women and data was not available to make this comparison. A higher total dose of synthetic oxytocin for labour augmentation (mean 1.3 IU) was reported to be significantly correlated with higher basal oxytocin levels (*p* = 0.03). Details of the correlation were not provided, and epidural analgesia exposure was not reported in this correlation.

At 2 months postpartum, researchers measured plasma oxytocin levels in a single blood sample taken during a home visit in women with varying exposure to synthetic oxytocin in labour and postpartum. Data was reported in two publications with large and seemingly overlapping populations, but with somewhat different analysis [53, 63]. Neither study compared oxytocin levels in exposed vs. unexposed women.

Prevost (2014) included only synthetic oxytocin administered by intravenous infusion for labour augmentation, with the total synthetic oxytocin of approximately 0.58–3.02 IU (mean total doses reported in four groups, extracted from Table 2 in the publication) [63]. In contrast, Gu (2016) included synthetic oxytocin administered both by labour infusion (2–20 mU/min) and postpartum by intramuscular injection, stated as 50 IU [53]. The total synthetic oxytocin dose was reported as 36 IU. Analysis was performed using ELISA in both studies. Both studies reported significant positive correlations between maternal basal plasma oxytocin levels and synthetic oxytocin total dose, which accounted for 2.2% of the variance in maternal oxytocin levels in Gu (2016). Gu (2016) also found more self-reported depressive, anxious, and somatisation symptoms in women with higher dose-exposure to synthetic oxytocin, which accounted for 4.7% of the variance in these symptoms.

##### Comments: synthetic oxytocin with later postpartum sampling: combined exposed-unexposed analysis

The low intrapartum infusion rates in these two studies would not be expected to raise maternal plasma oxytocin significantly above physiological levels, according to data in this review. Longer-term direct impacts on basal oxytocin levels are therefore biologically unlikely. This is also suggested by the small influence (2.2%) of synthetic oxytocin on variance in maternal oxytocin levels. Note also that a single blood sample may not be a reliable measure of maternal plasma oxytocin levels, which varied 70-fold, as reported in Gu (2016).

In relation to postpartum dosage, Gu (2016) reported a non-standard intramuscular dose of 50 IU, which is significantly higher than usual practice (5–10 IU) or manufacturer recommendations [29].

In addition, the reported 25-fold difference in synthetic oxytocin exposure was associated with similar maternal plasma oxytocin levels at 2 months postpartum, with means of 281.02 pg/mL (Prevost) and 286.3 (Gu). The reported influence on maternal mental health symptoms was small (4.7%), suggesting that factors other than synthetic oxytocin exposure may be more influential.

### Administration of maternal synthetic oxytocin and newborn plasma oxytocin levels

Six studies were identified that measured oxytocin levels in the umbilical cord blood of newborns whose mothers were administered synthetic oxytocin [44, 58–60, 62, 67]. (Table 4) Five studies involved intrapartum infusions of synthetic oxytocin, including one study with buccal as well as intravenous synthetic oxytocin [44]. One study measured newborn levels following maternal intramuscular injection at the birth of the baby [67].

All six studies separately reported the results from the umbilical artery (UA), which carries blood from fetus to placenta, and umbilical vein (UV), which carries blood from placenta back to the fetus. All studies also included newborns whose mothers did not receive synthetic oxytocin. In three studies, maternal plasma oxytocin levels were also measured in late labour, allowing comparison with newborn levels [58, 59, 62].

#### Newborns of women administered synthetic oxytocin infusions in labour

##### Newborn UA vs. UV levels

Levels of oxytocin in the UA were higher than in the UV in all newborns of women without synthetic oxytocin exposure. The same pattern was also seen in four of the five studies of newborns of women with intrapartum synthetic oxytocin exposure [58–60, 62]. However, Dawood (1978) found an inverse relationship, with UV higher than UA [44]. This reflected a much lower UA oxytocin compared to the control group, as discussed below.

##### Newborns of exposed vs. unexposed women

Of the studies that compared oxytocin levels (UA or UV) between newborns of women who had received or not received synthetic oxytocin in labour, four of five studies found no significant differences [58–60, 62]. However, Dawood (1978) found much lower UA oxytocin in newborns whose mothers received synthetic oxytocin (24.6 pg/mL) compared to levels in newborns of unexposed women (116 pg/mL) [44].

##### Newborn vs. maternal levels

Three studies measured oxytocin levels in late labour in women with and without infusions of synthetic oxytocin, as well as in their newborns [58, 59, 62].

In relation to maternal levels, Otsuki (1983) reported maternal plasma oxytocin levels in second stage labour in women who were administered synthetic oxytocin (infusion rate not stated) that were more than double the levels in unexposed women (*p* < 0.001) [58]. However, newborn oxytocin UA or UV levels were not different in the newborns of these exposed women vs. unexposed women in this study.

Padayachi (1988) and Pochard (1986) both reported oxytocin levels in women administered infusions of synthetic oxytocin that were not significantly elevated above controls. (Padayachi: 10.1 pg/mL with synthetic oxytocin vs. 9.1 pg/mL without; Pochard 3.0 vs. 2.0 respectively.) Rates and duration of synthetic oxytocin infusions were not reported but expected to be in a similar range to other included studies. In both studies, newborn UA and UV oxytocin levels were not different in the newborns of exposed vs. unexposed women [59, 62].

##### Anencephalic newborns

Of interest, Otsuki (1983) also included four women without synthetic oxytocin exposure who gave birth to newborns with anencephaly, a congenital condition that includes the absence of oxytocin-producing brain areas [58]. Newborn UA and UV oxytocin levels were below the level of detection, consistent with no oxytocin production, while maternal oxytocin levels were similar to levels in women with unaffected babies in this study.

#### Newborns of women administered postpartum intramuscular synthetic oxytocin

Sellers (1981) compared oxytocin levels in newborns of women who were exposed vs. unexposed to postpartum synthetic oxytocin by intramuscular injection (5 IU combined with 500 μg ergometrine with birth of the anterior shoulder) [67]. The timing of cord blood sampling following the injection was not stated. As in other studies, UA was significantly higher than UV (*p* < 0.05). Newborn UA and UV oxytocin levels were not different in the newborns of exposed vs. unexposed women.

#### Comment: newborn levels 

In these studies, newborn oxytocin levels were consistently higher than maternal levels, with the levels in the UA higher than the UV, which suggests fetal production of oxytocin in labour. Maternal infusions of synthetic oxytocin in labour did not influence newborn oxytocin levels. Anencephalic newborns were found to have undetectable oxytocin levels despite normal maternal levels, further suggesting that maternal oxytocin does not cross to the fetus in labour.

## Discussion

Synthetic oxytocin is very commonly administered to parturient women in current maternity care settings. This review is the first to summarise the effects of maternal intrapartum and postpartum administration of synthetic oxytocin on plasma levels of oxytocin in women and their newborns.

In these studies, maternal plasma oxytocin levels increased moderately in response to perinatal synthetic oxytocin infusions, with a dose-response pattern. Infusion rates of 10 mU/min or higher raised maternal oxytocin levels above the range seen in physiological labour. At maximum infusion rates (up to 32 mU/min), maternal oxytocin levels were 2–3-fold elevated compared to women in physiological labour. Newborn levels of oxytocin were higher than maternal and were higher in the umbilical artery than the umbilical vein, suggesting fetal production in labour. Oxytocin levels in newborns whose mothers received synthetic oxytocin in labour or postpartum were not higher than levels in newborns of unexposed women, suggesting that synthetic oxytocin is not transferred to the fetus in labour. Postpartum doses and maternal oxytocin levels were generally higher, but of shorter duration, compared to labour. Data analysed with RIA, especially studies involving serial samples, gave generally more coherent results than those using ELISA.

### Study design and other methodological considerations

The studies in this review are exploratory studies describing pharmacological and biological responses to the administration of synthetic oxytocin during the major physiological processes of labour and birth, rather than randomised controlled trials to ascertain the outcome of a clinical intervention. However, these included studies are of high quality and are very valuable in illustrating pharmacological and physiological processes.

Labour is a long biological process with an unpredictable rate of progress and is therefore difficult to study. There is a high variability in individual endogenous oxytocin levels, partly due to the pulsatile release of oxytocin during labour [1]. This makes it necessary to take multiple blood samples, which involves ethics and expense, especially for high-quality RIA assays. The majority of included studies involve repeated samples analysed with the same well-validated RIA technique. (See previous systematic review for a more thorough discussion of methodological considerations in relation to plasma oxytocin sampling in labour [1]).

In relation to randomisation, it is difficult to standardise the administration of synthetic oxytocin through the course of labour because infusion rates must be individually adjusted to accommodate clinical factors such as labour progress, pain, and fetal tolerance. Co-interventions such as epidural analgesia may also impact maternal plasma oxytocin levels in labouring women [77]. (The effects of epidural analgesia on maternal and newborn plasma oxytocin levels will be reported in an upcoming systematic review.) Therefore, a randomised trial of synthetic oxytocin at different dosages in labouring women would be problematic and unlikely to provide cohesive data. It is noteworthy that the only study in this review in which dosage of synthetic oxytocin was randomised occurred in the well-controlled post-caesarean context [73].

### Intrapartum synthetic oxytocin: maternal dose and oxytocin assays

The studies identified in this review are generally older, but the dose-regimens and infusion rates are similar to those used in current maternity care, as discussed below. This makes the maternal plasma oxytocin levels obtained in this review relevant to contemporary settings.

In the reviewed studies, starting rates were 1–3 mU/min, increasing to maximum 32 mU/min. Total doses of synthetic oxytocin administered during labour, reflecting infusion rate and duration, ranged from 0.5–15 IU, as stated in the studies or calculated from the data, based on maximum infusion rates continued for 8 hours (see Table 5). These calculated values are likely overestimated, as infusions are usually started at low rates and titrated upwards only when necessary.

Similarly, a recent study that analysed contemporary intrapartum dose regimens from 13 mostly European countries found starting infusion rates varying from 1 to 15 mU/min, with maximum rates between 15 and 30 mU/min in most regimens [78]. The highest maximum infusion rate was 60 mU/min, based on high-dose regimens in two countries. The authors of this study also calculated a total intrapartum dose of 2.38–27 IU (mean 8.97 IU), based on a theoretical 8-hour infusion and incorporating the starting rate, interval for rate increases, and maximum infusion rate. Again, these calculations are likely overestimations of the total doses used in clinical practice because infusion rate increases are titrated against clinical effects.

Most of the older studies included in this review analysed oxytocin levels with radioimmune assay (RIA), which remains the gold standard assay for oxytocin analysis. The RIA studies in this review showed consistency in both dose-response relationships and in comparisons between levels obtained in physiological birth and with synthetic oxytocin infusions. This allowed clear comparisons between studies and consistent interpretation of the data.

In contrast, most of the more recent included studies have used enzyme-linked immunoassays (EIA, ELISA), which are less specific for the oxytocin molecule compared to RIA, and the numerical values obtained with ELISA are higher and less consistent [38, 76]. For example, Ende (2019) found no rise in maternal plasma oxytocin using ELISA at one-hour post-caesarean in response to intraoperative intravenous administration of (mean) 14 IU synthetic oxytocin, whereas Yamaguchi (2011), also using ELISA, found 10-fold elevation at 1 hour post-caesarean following 10 IU infused over 30 minutes [47, 73]. Included studies that have reported results from a single sample and also used ELISA for analysis are particularly difficult to interpret [53, 63].

The trend towards the use of ELISA in more contemporary maternity-related studies, compared to the “well validated, but more laborious” RIA [38] used in the older studies, may have fostered misconceptions regarding oxytocin levels in response to synthetic oxytocin and the possibility of direct biological impacts on mother and/or offspring. Models of oxytocin physiology have been developed that are not anchored in the original, detailed knowledge and understandings of oxytocin release in childbirth and the pharmacological effects of synthetic oxytocin, as seen from the RIA studies this review.

In particular, the pharmacokinetic properties of synthetic oxytocin remain poorly understood, despite its widespread administration to labouring women.

### Synthetic oxytocin: pharmacokinetics

Older data, based on labelled (tritiated) synthetic oxytocin (5 IU) administered intravenously mid-pregnancy, and on in-vitro measurements, suggested a half-life of 5 minutes in pregnant women [79, 80]. One included study by Gibbens found a half-life of 3 minutes following postpartum synthetic oxytocin intravenous bolus (5 IU), with a considerably slower decline after this initial rapid decrease [52]. However, steady-state calculations (discussed below), including in the reviewed studies by Seitchik (1984) and Amico (1984), suggest a longer half-life of 8–10 minutes for synthetic oxytocin, at least in labouring women [42, 65, 81].

Outside of pregnancy, Amico calculated a half-life of 12–17 min (mean 15.3) in four men administered synthetic oxytocin infusion at 125 mU/min [42]. In other high-quality data, Legros and colleagues found a slower decline in men following 8 mU/min infusion, with data suggesting a half-life of around 20 mins [82]. In a very detailed pharmacokinetic study, Nielsen administered synthetic oxytocin (10 IU bolus) to postmenopausal women and reported a more complex 2-compartment model of distribution, with half-lives of 5.5 minutes and 1.2 hours respectively [83].

The half-life could be shorter in labour due to enhanced placental production of a specific oxytocinase, placental leucine aminopeptidase (P-LAP). This enzyme rapidly degrades oxytocin and enzyme levels are increased up to 10-fold in late pregnancy [84]. In one included study, Thornton and colleagues found a four-fold lower plasma oxytocin response to synthetic oxytocin dosage and a higher metabolic clearance rate (MCR) in pregnancy vs. postpartum, suggesting effects from P-LAP, although half-life was not calculated [70]. Others have not found differences in MCR in pregnant vs. non-pregnant context [85].

Synthetic oxytocin product information sources give wide ranges of values for oxytocin half-life (e.g. 3–20 minutes), reflecting this lack of precise data [29, 86].

Steady state is another important pharmacokinetic concept. This reflects the time from a dose increase until ‘steady state’ is reached: that is, when input and degradation are balanced. Steady state is generally 3–4 times the half-life, according to basic pharmacokinetic rules. If dose levels are very high and metabolism cannot keep pace with input, drug accumulation will occur. There was no evidence in the included studies for accumulation of oxytocin at the doses of synthetic oxytocin used.

In relation to synthetic oxytocin, both Amico and Seitchik found that steady-state was reached at 30–40 minutes after an increase in infusion rate, which is similar to steady-state calculations performed by Dawood, and fits with a half-life of 8–10 minutes [42, 65, 81]. Similar estimates of half-life and steady state are provided by more contemporary sources [87–89]. This suggests that it may be more effective to gradually increase synthetic oxytocin infusion rates and titrate this against individual uterine effects, using intervals of 30–40 minutes.

The consistent dose-response curves and other pharmacokinetic data across the included studies show a linear increase in oxytocin levels with each increase in the infusion rate (Table 3). These findings are also consistent with manufacturer data and recommendations, and similar data has been reported outside of pregnancy [29, 82, 83].

It is also interesting to note that early clinicians saw benefit in using the lowest infusion rate possible and staying within the range of physiological oxytocin levels, which this data suggests is 9 mU/min or less. Similarly, lowering maximum infusion rate to 10.9 mU/min was found to improve induction outcomes in a recent US-based quality improvement project [90]. Clinicians have also trialled a pulsed infusion of synthetic oxytocin for induction or augmentation, which was found to substantially reduce the total dose administered without loss of efficacy in most studies, and may deserve further exploration [81, 91–95].

### Pharmacokinetics and clinical use

According to the physiological model, as presented in Fig. 1, the infusion rate and the ensuing plasma oxytocin levels will influence the uterine response (and subsequent metabolic, autonomic and hemodynamic effects) more than the total amount (total dose) of synthetic oxytocin. (Note that the total dose may be high when infusions are prolonged, but the rate of infusion may still be low.)

It is also important to note that the sensitivity of the pregnant woman’s uterus to oxytocin increases substantially towards term, due to increases in oxytocin receptors and other activating processes [7, 96, 97]. According to one included study, the amount of synthetic oxytocin required to stimulate the uterus and augment labour can vary more than 5-fold, due to differences in sensitivity of the uterus and oxytocin receptors [42].

In addition, there is a biological limit to the infusion rate of synthetic oxytocin that can be safely administered in labour. An excessively high infusion rate will cause uterine hyperstimulation, with reduced blood flow that increases risks of hypoxia for the fetus and maternal metabolic and autonomic consequences as described in the physiological model (Fig. 1). The safe maximum infusion rate varies according to the reactivity of the individual woman’s uterus and the tolerance of her fetus to the effects of contractions, among other factors [98]. The maximum rate found in the included studies was 42 mU/min*.* Similarly, the highest recommended maximum infusion rate is 40 mU/min in contemporary protocols for active management of labour and 40–60 mU/minute in EU guidelines [78, 99].

### Postpartum synthetic oxytocin

In contemporary maternity-care, synthetic oxytocin is commonly administered by intramuscular injection (5–10 IU) to prevent postpartum haemorrhage (PPH) after vaginal birth. It may also be administered by intravenous infusion as a treatment for PPH following vaginal birth, or routinely following caesarean section. Postpartum infusions are generally administered at much higher rates than intrapartum infusions but are administered for a more limited time. The total postpartum dose (by intravenous infusion or intramuscular injection) following vaginal birth may therefore be similar to the total dose that is given over the duration of labour, as seen in several of these studies.

More recently, an intravenous bolus of synthetic oxytocin has been advocated as a more effective PPH preventative following vaginal birth than intramuscular administration [100, 101]. A slow intravenous bolus of 10 IU is now recommended following vaginal birth, where feasible, by the World Health Organisation [36].

In this review, only one study was found that measured maternal plasma oxytocin levels following a postpartum intravenous synthetic oxytocin bolus (5 IU). Gibbens and colleagues (1972) found plasma oxytocin peaks up to 700 pg/mL in the first minute after administration [52].

Outside of maternity care, the pharmacokinetic study discussed above found plasma oxytocin peaks around 1300 pg/mL at 15 minutes, more than 70-fold above basal levels (17 pg/mL), in response to a 10IU bolus [83]. Peak levels would have been higher if samples had been collected immediately after injection. Oxytocin levels declined more slowly to basal over two to four hours, showing a similar two-phase disappearance to that found by Gibbens.

Given these high oxytocin levels in response to bolus intravenous injection, potential adverse effects should be considered when administered to women postpartum. Of particular concern, researchers have identified significant hemodynamic disturbances (well-recognised side effects of synthetic oxytocin) after administering 2.5–10 IU by intravenous bolus to healthy women following pre-labour caesarean. Some women have had indications of coronary vasoconstriction, likely because of the rapid oxytocin peaks in combination with the hemodynamic disruptions of regional anaesthesia [102–107]. These adverse effects may be less significant following vaginal birth, but they are an indication of the very high levels of oxytocin following intravenous bolus. The possible hemodynamic impacts of bolus intravenous synthetic oxytocin following vaginal birth in combination with epidural analgesia, or in women with cardiovascular vulnerabilities, may also require consideration before widespread adoption of routine postpartum intravenous bolus.

### Newborn plasma oxytocin levels following maternal synthetic oxytocin administration

In this review, newborn oxytocin levels, as measured in cord blood, were consistently higher than maternal plasma oxytocin levels. In addition, oxytocin levels were consistently higher in the uterine artery (UA), which represents production by the fetus, than in the uterine vein (UV), which reflects processes in the placenta, including possible placental transfer and metabolism. These findings suggest that the human fetus has its own oxytocin production during labour and is consistent with understandings that the human fetus has a relatively mature oxytocin system at birth [108–111].

Lower UV oxytocin vs. UA also suggests that fetal oxytocin is metabolised in the placenta, likely by the high levels of placental oxytocinase, which may also contribute to reducing placental transfer [112, 113].

In this review, no increase in newborn oxytocin levels was reported following maternal administration of synthetic oxytocin in labour, even in newborns whose mothers received high infusion rates, and had high oxytocin levels, in late labour (Table 4). Of interest, in one included study, Otsuki (1983) found no oxytocin in the cord blood of anencephalic newborns, who lack the brain centres that produce oxytocin [58]. This further suggests that oxytocin in the cord blood derives from the fetus and that oxytocin, whether endogenous or synthetic, is not transferred across the placenta.

### Can synthetic oxytocin cross biological membranes?

These findings are in accord with biochemical understandings. Peptide molecules such as oxytocin are hydrophilic and do not readily cross biological membranes. It has been reported that 0.002–0.1% of plasma oxytocin can cross to the brain through the blood-brain barrier [114–116] and passage across the placenta is likely to be similarly limited. Significant transplacental passage would therefore require extremely high maternal levels (100-fold or more above physiological), which are not reached in labouring women administered synthetic oxytocin, according to this review. In addition, the P-LAP oxytocinase enzyme, produced by the placenta at the interface between fetal and maternal blood, may further prevent transfer of maternal oxytocin to the fetus by degrading the molecule on contact [117].

Direct studies of transplacental passage of synthetic oxytocin have had contradictory findings. Some of the conflicting data may be related to the experimental techniques used. For example, some in-vitro studies using human placentae have found a small and delayed transplacental passage, but placental oxytocinase and membrane barriers may be functionally reduced in this model [113, 118]. In addition, oxytocin levels in the maternal compartment (perfusate) in these models are significantly higher than the maternal levels found in this review in response to synthetic oxytocin administration [113].

Of note, a detailed in-vivo sheep model, which involved fetal and maternal instrumentation and direct blood sampling, did not show passage of an intravenous infusion of synthetic oxytocin from mother to fetus, or from fetus to mother [119].

### Dose-exposures in animal models

Offspring biological effects from perinatal synthetic oxytocin exposures have been demonstrated in animal models. These findings are often used as evidence to support the passage of synthetic oxytocin to the human fetus at clinically-relevant doses, and even to the fetal or newborn brain. However, to further understand the relevance of such animal studies to human synthetic oxytocin exposures, it is necessary to understand the comparative dose-exposures.

Animal dose-exposures can be calculated from published data and compared to the dose-exposures for women and newborns, as seen in this review. (See legend in Table 3 for oxytocin conversions.) For example, in the in-vivo sheep model discussed, the maternal infusion rates administered (0.8 and 0.08 mU/min per kilogram) correspond to clinically relevant infusion rates of 5.6–56 mU/min for a 70 kg (kg) pregnant woman and caused uterine contractions in the pregnant ewe. In this model, there was no passage of synthetic oxytocin from mother to fetus [119].

In other animal models, researchers have exposed prairie vole fetuses indirectly to synthetic oxytocin via maternal administration in late pregnancy, or directly administered synthetic oxytocin to newborns, and studied offspring neurobiology and development.

Dose-exposure calculations show that this animal data cannot be compared to human perinatal exposures because of the extremely high doses of synthetic oxytocin that are administered. Bolus doses administered to pregnant prairie vole dams of 0.03 to 0.5 mg (18–300 IU) per kilogram are equivalent to 1260–21,000 IU for a 70 kg pregnant woman [120]. Typical boluses administered to newborn voles of 0.03-3 mcg to 1-8 mg/kg to a 2g newborn are equivalent to 90-4800 IU per kg, corresponding to 720–14,000 IU administered to a 3 kg human newborn [121]. These doses are one hundred- to ten thousand-fold higher than the total doses that women receive in labour (Table 5). Such extreme doses could not be administered clinically because of the sensitivity of the human uterus to oxytocin, associated with the large increase in oxytocin receptors at term [7]. These doses would cause uterine hyperstimulation and endanger the fetus, as well as causing adverse maternal hemodynamic effects, as discussed.

However, these high animal-model doses of synthetic oxytocin will certainly produce very high plasma oxytocin levels, with even more extreme peaks with bolus administration. The likely 0.1% transplacental and blood-brain passage could undoubtedly induce biologically significant effects for the fetus or newborn. In these studies, vole offspring who received these exposures directly or indirectly via maternal administration had enhanced adult social and parental behaviours, compared to offspring of untreated dams, along with changes in brain oxytocin and other hormonal systems [120, 121]. These findings suggest positive biological effects on offspring oxytocin systems from very high-dose exposures in the perinatal period.

In other studies, rat dams administered similarly high doses of synthetic oxytocin in early pregnancy had less body fat at the end of pregnancy and gave birth to larger offspring with bigger placentas than untreated dams [122]. Newborn rat pups directly administered similar doses to newborn voles had more tolerance to stress and pain in adulthood compared to untreated offspring, indicating that the oxytocin system was positively modified by these high exposures [123, 124].

It has also been suggested that synthetic oxytocin might cross the maternal blood-brain barrier in labour or postpartum and directly impact central maternal oxytocin functioning, including possible associations with maternal postpartum mental health [125, 126].

This model has several limitations. As noted, 0.1% of plasma oxytocin is estimated to cross into the brain. This review found that the doses of synthetic oxytocin administered in labour cause maternal plasma oxytocin elevations 2–3-fold above levels in physiological labour, which would be insufficient to cross to the brain and influence central processes.

In support, primate research found that synthetic (labelled) oxytocin administered by intravenous bolus does not cross into brain tissue, even at high bolus doses of 40-80 IU. These large boluses produced very high plasma levels (mean 1992 ± 476 pg/ml) and small elevations in CSF (below the level of quantification) as predicted by low passage through the blood-brain barrier [127].

Animal studies have suggested an active transport mechanism into the brain using RAGE (receptor for advanced glycation end- products), based on high bolus doses of synthetic oxytocin (e.g. 0.65 IU/kg) which could not be safely administered to women in labour. At such doses, oxytocin could also pass directly into the brain via the blood-brain barrier. In addition, afferent stimulation of sensory nerves by peripheral oxytocin could also promote central oxytocin release [128, 129].

For these reasons, it is not accurate to compare the plasma levels, or the effects, of infusion of microunits of synthetic oxytocin, as used in clinical maternity care, with animal studies administering milligram doses. At these high milligram doses, passage through membranes can occur, but this does not occur at the lower milliunits exposure that occurs in human labour and birth. Therefore, the data in this review do not support direct maternal or offspring biological effects, short- or longer-term, from the direct passage of synthetic oxytocin administered in labour or postpartum into the maternal brain.

Direct epigenetic effects on the oxytocin system have been demonstrated in animal offspring in response to very high peripartum exposures to synthetic oxytocin [120]. However, such effects are unlikely to occur for women and human offspring at the comparatively low dose-exposures and resulting low maternal oxytocin levels found in this review. In addition, a recent review found no evidence, thus far, of epigenetic effects from physiological childbirth in human studies and suggested, “oxytocin linked effects might be indirectly mediated via other receptors and signalling systems.” [130].

### Alternative, indirect mechanisms for synthetic oxytocin biological effects

Some studies have suggested negative maternal and infant outcomes associated with maternal synthetic oxytocin administration, including reduced newborn pre-breastfeeding behaviours; delayed breastfeeding initiation; and shorter duration [131–137] (see Erickson (2017) for recent review [138]). Maternal postpartum mood and wellbeing has also been suggested to be negatively impacted, although not all studies have found negative maternal mental health outcomes [53, 139–141]. (See also Kendall-Tackett for critique [142]). Such possible adverse effects may reflect indirect influences of synthetic oxytocin on mother and baby, including via changes in the quantity and quality of uterine contractions.

### Possible indirect effects: maternal

Infusion of synthetic oxytocin in labour at clinically high doses causes stronger and more frequent uterine contractions, with shorter periods of relaxation between contractions, compared to physiological labour [29–31]. Strengthening of contractions exacerbates the metabolic effects within uterine muscle (illustrated in Fig. 1) including by further reducing blood flow, increasing lactic acid levels and reducing pH. In addition, reduced intervals between contractions leaves less time for recovery within the uterine muscle, increasing the metabolic stresses. Sensory input from the uterus to the brain in response to these stresses generates pain sensations and shifts autonomic function further towards the stress-linked sympathetic nervous system (SNS). The release of endogenous oxytocin may be decreased as the contraction proceeds, due to SNS feedback [20]. (Note that this would be masked by synthetic oxytocin.) See Background for further details.

The processes of labour may eventually slow due to these metabolic, autonomic and possibly oxytocin-reducing effects. Doses of synthetic oxytocin are often increased to compensate this decline, which further exaggerates these metabolic stresses and consequences.

Note that perinatal synthetic oxytocin administration may influence endogenous oxytocin production or levels both negatively and positively. Negatively, as discussed and shown in Fig. 1, oxytocin levels can be reduced due to SNS feedback in response to strong contractions. Positively, pressure on the cervix induces sensory feedback, which augments endogenous oxytocin levels via the Ferguson reflex. It is also possible that synthetic oxytocin might stimulate activity of the vagal nerve or other sensory nerves thereby stimulating central oxytocin release. By these mechanisms, synthetic oxytocin may, in some circumstances, stimulate some of the beneficial effects of endogenous oxytocin that occur during physiological labour and birth. (See Background for more details.)

This is consistent with positive effects for synthetic oxytocin reported in some circumstances. In one included study, Velandia (2012) reported that, following pre-labour caesarean, only those women who received postpartum 50 IU of intravenous synthetic oxytocin (in addition to standard 5 IU) released significant oxytocin during skin-to-skin contact with their newborns. Those women who received synthetic oxytocin also reported more positive personality changes at 2 days postpartum, including the lowest scores on detachment and somatic anxiety, compared to women without synthetic oxytocin [72]. (The effects of caesarean section on maternal and newborn plasma oxytocin levels will be reported in an upcoming systematic review.)

Positive effects have also been reported in relation to synthetic oxytocin administration with vaginal birth. In one included study, Handlin (2009) and Jonas (2009) found lower stress and anxiety and greater sociability in women with vs. without infusions of synthetic oxytocin in labour at very low doses (mean total dose 1.6 IU) [54, 143]. Similarly, a more recent study found more positive postpartum personality traits in women with vs. without synthetic oxytocin, administered in labour at a (median) maximum infusion rate of 7.5 mU/min [144].

These potential benefits of synthetic oxytocin may especially apply when the endogenous oxytocin system has not been activated, such as following prelabour caesarean, although research is limited [145, 146]. (See Jonas 2009 for further discussion [57].) Adverse impacts of synthetic oxytocin due to other mechanisms or co-interventions also need to be considered, as discussed above.

Whether synthetic oxytocin has harmful or beneficial impacts likely depends on the intensity of uterine contraction patterns. Stronger and more frequent contractions caused by synthetic oxytocin can increase physiological stresses that may increase SNS activation and shift the ANS balance towards the SNS and stress (Fig. 1). Longer-term negative impacts by such contractions on maternal physiology are biologically plausible.

### Postpartum administration: possible indirect effects

The pharmacological effects of synthetic oxytocin on maternal physiology will differ when administered postpartum compared to intrapartum. Postpartum contractions are shorter in duration and less frequent compared to intrapartum contractions, even when synthetic oxytocin is administered postpartum [147, 148]. These relatively shorter and less frequent contractions give more time, within the uterine muscle, for acidity and lactic acid to clear and tissue stresses to reduce. Adverse impacts via maternal metabolic and autonomic effects including SNS activation (Fig. 1) are therefore less likely following postpartum vs. intrapartum synthetic oxytocin.

However, the dosage and route of administration of postpartum synthetic oxytocin, and the resulting oxytocin levels and patterns will also be important factors. Adverse haemodynamic or other effects are also possible. (See ‘Pharmacokinetics and clinical use’, and ‘Postpartum synthetic oxytocin’). Note also that other oxytocic drugs including ergometrine and prostaglandins may have different pharmacological effects in the postpartum period, including possible adverse effects on lactation hormones [149, 150].

### Synthetic oxytocin and co-interventions

Investigation of possible maternal impacts from synthetic oxytocin may also require consideration of epidural analgesia, which is commonly co-administered to assist women with increased pain from the stronger contractions [151, 152]. Epidurals may reduce the sensory feedback that drives the Ferguson reflex, causing oxytocin levels to decrease and contractions to slow [77, 153–155]. To compensate, higher doses of synthetic oxytocin are often required [152].

In one included study, Jonas (2009) measured plasma oxytocin levels during early breastfeeding and found the lowest levels among women who received both synthetic oxytocin and epidural analgesia in labour [57]. A recent re-analysis of the data from this study has reinforced these findings, reporting no negative impacts on oxytocin release during early breastfeeding in women administered synthetic oxytocin in labour alone; that is, without concomitant epidural analgesia [68].

In addition, epidurals reduce the sensitivity of sensory nerves for both mother and newborn, potentially uncoupling the physiological interactions (mutual regulation) that guide mother and baby after birth and causing a ‘pharmacological separation’ [4]. (The effects of epidurals on maternal and newborn plasma oxytocin levels will be reported in an upcoming systematic review.)

### Induction vs. augmentation

There may also be different maternal physiological effects from administration of synthetic oxytocin for labour augmentation vs. induction. In the present review, infusion rates and total doses were generally higher for induction than for augmentation, likely reflecting the reduced responsivity of the uterus before the physiological onset of labour, including fewer oxytocin receptors [7].

Longer-term effects on maternal psychological wellbeing have been reported following induction of labour [156–158]. This could relate to deficits in the full readiness of central oxytocin and other maternal systems. Some biological aspects of maternal-newborn bonding could also be affected, but human research is limited. In animal studies preparatory changes in the maternal brain may only occur in the last stages of pregnancy [159].

Induction also involves foreshortening of gestation, including of the full period of fetal brain development, with accumulating evidence of neurodevelopmental and educational deficits at earlier vs. later gestations, even up to 40-41 weeks [160–167].

It is also important to note that induction studies have generally not differentiated the method of induction (synthetic oxytocin, prostaglandins, mechanical methods), which may impact the consequences of induction. (The effects of prostaglandins on maternal and newborn plasma oxytocin levels will be reported in an upcoming systematic review.)

### Oxytocin receptor considerations

Prolonged synthetic oxytocin administration in labour may lead to reduced uterine responsiveness and has been identified as a risk factor for postpartum haemorrhage in many studies [168–173]. It has been suggested that these well-recognised clinical effects reflect desensitisation (reduced binding) and possibly down-regulation (reduced numbers) of uterine oxytocin receptors [174, 175].

It is unlikely that classical receptor desensitisation would occur in vivo in response to synthetic oxytocin administration in labour. As identified in this study, clinical doses produce relatively modest maternal oxytocin plasma levels (in the picomolar range). Researchers have determined that oxytocin receptor desensitisation occurs in vitro from 1 nanomole (nM) exposure, equivalent to 1000 pg/mL (see conversions in Tables 3 and 4) with maximal desensitisation at 1 μmol (μM) equivalent to 1,000,000 pg/mL [174, 176, 177]. These levels are 10- to 10,000-fold higher than levels measured in labouring women with high-dose synthetic oxytocin infusions, according to this review. Other in vitro research has found similarly high thresholds, with oxytocin receptor desensitisation only occurring at tissue oxytocin exposures above 10^− 8^ M, equivalent to 10,000 pg/mL [178]. In addition, the desensitisation effects in these studies begin after 6 hours exposure and peak at 20 hours, which is a relatively long duration for synthetic oxytocin infusion.

Of note, these researchers also found reductions in receptor binding and density with the progress of physiological labour [174]. This may be due to the metabolic and autonomic effects described, including lactic acid build up and reduced pH within uterine tissues (Fig. 1), which could also reduce receptor functioning [17, 19]. This raises the possibility that the reported negative effects of synthetic oxytocin on oxytocin receptors may represent an amplification of the metabolic effects of physiological labour, including greater increases in uterine lactic acid and acidity (Fig. 1). Such metabolic effects would occur more quickly than the classical desensitisation effects seen in vitro and will be greater with longer periods of infusion.

In support, researchers have shown benefits to labour outcome and newborn wellbeing in women diagnosed with labour dystocia from prior oral bicarbonate treatment [179]. This alkalinising treatment counteracts the lactic acid build up and pH reduction due to excessive uterine muscle activity (see Fig. 1), thereby improving myometrial function, likely including oxytocin receptor function. This metabolic model could account for some of the clinical effects of prolonged synthetic oxytocin administration, including the reduced effectiveness with long duration and the increased risks of postpartum haemorrhage.

Whether the negative effects of synthetic oxytocin on uterine activity reflect direct effects on oxytocin receptors or indirect metabolic effects that might also reduce receptor functioning, limiting the use, and especially dose, of synthetic oxytocin is likely to provide benefits for labouring women and their babies.

### Possible indirect effects: fetus/newborn and offspring

For the offspring of women exposed to synthetic oxytocin in labour, researchers have suggested increased longer-term risks of adverse neurodevelopmental sequelae, including attention-deficit hyperactivity disorder (ADHD) and autism, compared to offspring of unexposed women [180–189]. Much of the evidence thus far for these negative outcomes is low-quality and differences are smaller or completely negated in large or more detailed studies or reviews [126, 190–194].

As discussed, this review of studies measuring oxytocin levels in labouring women and newborns found no evidence that synthetic oxytocin, at clinical doses, crosses the placenta and elevates fetal/newborn oxytocin levels. In fact, the high newborn oxytocin levels found in all relevant studies in this review, including in newborns of women without synthetic oxytocin exposure, suggest that the human fetus is actively producing oxytocin in labour. Direct physiological effects of maternal synthetic oxytocin on the fetal oxytocin system are therefore extremely unlikely.

However, indirect effects are possible for the fetus/offspring, as for the mother, likely via changes in contraction patterns. Synthetic oxytocin causes contractions that are both stronger and more frequent, compared to contractions in physiological labour [30, 31, 195, 196]. Both factors contribute to a greater reduction in blood flow to the fetus, giving exaggerated metabolic effects, compared to labour without synthetic oxytocin (Fig. 1).

Stronger synthetic oxytocin-driven contractions cause increased pressure on the placenta, reducing placental blood flow to the fetus during contractions. Researchers using Doppler ultrasound found 50% reduction in placental blood flow during contractions in women administered synthetic oxytocin or prostaglandins for labour induction, compared to 33% reduction in women during spontaneous labour. Blood flow was also reduced between contractions, although no adverse effects were detected on fetal heart rate monitoring in this study [197].

The higher frequency of contractions due to synthetic oxytocin can also be detrimental for the fetus in labour, disrupting the balance between contraction and relaxation and reducing the relative period of relaxation and recovery between contractions (Fig. 1).

Both increased strength and frequency of contractions, driven by synthetic oxytocin, can overwhelm the normally adequate fetal adaptations to labour hypoxia, especially for the vulnerable fetus [25, 197, 198].

Detailed studies using an in-vivo model in sheep have found that the fetus can withstand extreme hypoxia (intermittent umbilical cord occlusion for 1 minute) if there is sufficient time for reperfusion and recovery [199, 200].

Clinical researchers have observed that complete post-contraction recovery of fetal heart rate and fetal oxygen saturation requires intervals of at least 2 minutes between contractions and that relatively shorter intervals increase the risk of low pH in newborn cord blood (acidaemia) [201–203]. Similarly, markers of fetal cerebral hypoxia in labour, which correlates with newborn acidaemia, are worsened when the interval between contractions is less than 2.3 minutes [204, 205]. A greater degree of newborn acidaemia predicts higher morbidity, including lower APGAR scores and higher risks of respiratory morbidity and newborn intensive care unit (NICU) admission [206].

These considerations highlight the potential hazards of synthetic oxytocin for the fetus/newborn. Newborns whose mothers receive synthetic oxytocin infusions in labour, compared to newborns of women without synthetic oxytocin, have increased risks of acidaemia; NICU admission; hypoxic-ischaemic encephalopathy, a marker of brain compromise; convulsions and other indications of neurological morbidity; and in some settings, increased risks of neonatal death [207–212]. These risks are highlighted by the Institute for Safe Medication Practices, who nominated synthetic oxytocin as a high-alert medication that carries “a heightened risk of harm” and requires “special safeguards to reduce the risk of error” [213]. According to Clark (2009), allegations of synthetic oxytocin misuse may be involved in half of all paid obstetric litigation claims [214]. For these reasons, monitoring of fetal wellbeing, including fetal heart rate monitoring, is essential when synthetic oxytocin is administered in labour.

Newborns of women administered synthetic oxytocin in labour also have higher risks of jaundice, likely due to increased erythrocyte fragility [215–218]. Studies have also found that newborns of exposed women have lower levels of glutathione and markers that indicate more oxidative stress, compared to newborns of unexposed women, likely due to hypoxic effects [219–221].

As discussed, studies have also linked maternal exposure to synthetic oxytocin in labour with changes to newborn breastfeeding behaviours and with reduced breastfeeding success and duration in most studies [131–136]. Some findings suggest an earlier onset of pre-breastfeeding behaviours [137]. (See Erickson (2017) for review [138]). The mechanisms for such possible effects are not clear and may involve impacts in both mother and baby, as discussed above. Epidural analgesia may be a confounding factor for negative impacts, and induction of labour (by any method) may also impact breastfeeding, possibly by pre-empting full newborn readiness [138, 222]. Disruptions to breastfeeding can have significant detrimental long-term impacts on offspring health and wellbeing [223, 224].

Another possible mechanism for indirect offspring impacts is an increased risk of maternal-newborn separation due to co-interventions such as epidural and caesareans, which may also impact breastfeeding success [225–227].

In summary, synthetic oxytocin administration in labour makes uterine contractions stronger and more frequent, compared to physiological birth. At higher infusion rates, this can increase fetal hypoxic stresses, with well-recognised risks for the fetus and newborn. Longer-term offspring impacts are also possible.

### Strengths and limitations of this review

There are strengths and limitations in the studies included in this review. This review, like all systematic reviews, can only make conclusions based on the existing publications, as identified according to the chosen research question and search terms.

It is likely that the vast majority of relevant studies would have been published in peer-reviewed journals and identified in the chosen data bases using the configured search terms. Negative publication bias is unlikely, as the data is neutral in relation to hypotheses and models. In addition, while a preponderance of older studies may seem a limitation, the older studies have generally provided the most reliable and consistent data, including the use of the gold-standard RIA assay. Some of the data in these older studies is unique and no longer obtainable, as similar studies cannot be performed today for ethical, practical and/or funding reasons.

The women included in these older studies may have some different characteristics to modern populations, who may be older or with higher BMI. However, the pharmacological responses to synthetic oxytocin, as documented here, would not be expected to vary substantially between populations. Of interest, one included study (De Tina 2019) found no difference in oxytocin levels in response to synthetic oxytocin infusions in obese vs. non-obese women [46]. Some studies have found longer labour, and longer duration of synthetic oxytocin infusions in women with higher BMI, but generally not higher infusion rates [228, 229].

One limitation is that relatively few studies were identified, especially in some categories, making the small amount of data in these areas less robust. In addition, some studies did not clearly define dose, administration, and sample timings, and control groups were not always included.

A major strength of this review and methodology is the collating of all available numerical data on maternal and newborn oxytocin levels in response to maternal synthetic oxytocin administration. This has facilitated data-based pharmacological calculations of infusion rates, the total doses administered to labouring women and the resulting oxytocin levels. This data can then be compared with data from other clinical studies, and with levels in physiological labour. These comparisons can also inform clinicians in establishing thresholds in dosage and pharmacological effects that may exceed physiological parameters and potentially over-ride maternal and/or fetal adaptations. The infusion rates seen in this review are similar to those recommended in modern maternity-care guidelines and protocols, as discussed.

This hard data and calculations also provide important and unique perspectives on comparative dose-exposures for animal offspring. Calculations in this review show that dose-exposures in current animal studies, whether administered to the pregnant female or to the newborn, are many-fold higher than safe human exposures for the pregnant woman or her fetus/newborn. This adds important caveats to the interpretation of current animal models of synthetic oxytocin exposure, including in relation to possible long-term impacts. These considerations are also underlined by the included human data on newborn oxytocin levels, which did not rise in response to maternal synthetic oxytocin administration.

This methodology and data are also uniquely valuable in assessing the likelihood of direct biological impacts of perinatal exposure to synthetic oxytocin on the maternal brain. Again, the hard data in this review, and calculations based on this data, do not support direct maternal psychological or neurological effects from synthetic oxytocin transfer into the maternal brain in the perinatal period.

Another strength of this review is the wide cross-disciplinary authorship, providing understandings and perspectives from many fields to widen the scope for data interpretation. This includes midwives, obstetricians, clinicians, a psychiatrist, and researchers with competence in physiology and pharmacology. This breadth has allowed a deeper understanding of physiological, pharmacological and clinical implications, including other mechanisms that could contribute to maternal and/or offspring impacts from peripartum synthetic oxytocin exposure.

In addition, the detailed physiological knowledge of oxytocin analysis and assay characteristics in this paper, together with precise descriptions and understanding of dose levels and concentrations will facilitate the use of synthetic oxytocin in safe and efficient ways by clinicians, and provide basic and accurate information for researchers and other interested parties. 

### Clinical implications

Given the large numbers of women who receive synthetic oxytocin in the perinatal period, it is critical to understand the clinical impacts for women and their offspring. The clinicians who prescribe and administer this drug must know exactly how much they are giving, in order to optimally adapt the dosage and reduce the risks of potentially serious side-effects for women and their babies. This review adds critical information about how these dose regimens might impact maternal plasma oxytocin levels, which is essential in understanding safety in the short and longer terms.

One important finding is that infusion rates below 10 mU/min are unlikely to raise maternal plasma oxytocin levels above physiological levels. (Note that only data obtained by the well-validated, specific and sensitive RIA can be used in these types of comparisons and analysis.) Based on this data, as far as possible “… the dose of oxytocin required to establish labour in term pregnancy should be low and within the physiological range.” (Dawood 95, p587) [81].

The data in this review also aid the reader in calculating how much synthetic oxytocin is given, facilitating comparisons with other clinical studies (Table 5). Such comparisons are often difficult due to different dose regimes and the translation from infusion rates in mU/min to total doses in IU. Calculations of dose-exposure also help readers to judge where animal research is relevant to clinical human studies.

In relation to concerns about synthetic oxytocin crossing directly into the maternal brain or to the fetus in labour, this review found that the doses of synthetic oxytocin used in clinical practice give only moderate elevations in maternal levels, which are insufficient to cross the maternal blood-brain barrier or to cross the placenta to the fetus in biologically significant amounts. Therefore, according to the data in this review, direct biological effects from infusions of synthetic oxytocin at clinical doses are extremely unlikely for women or offspring.

However, caution is urged in the administration of synthetic oxytocin in labour, as significant indirect impacts for women and offspring are recognised. For women, the stronger and more frequent uterine contractions driven by synthetic oxytocin will generally reduce uterine blood supply to a greater extent than during physiological labour, especially at higher doses. This deficit will inevitably increase hypoxia, lactic acid and physiological stresses within uterine tissues, which could shift maternal autonomic functioning towards stress and away from the oxytocin-related parasympathetic calm and connection physiology. If these effects become sustained, this could account for associations between the use of maternal synthetic oxytocin infusions and negative impacts on breastfeeding and maternal psychological wellbeing as discussed, but has not been well-researched.

For the fetus, the stronger and more frequent contractions driven by synthetic oxytocin will compress placental blood vessels and reduce fetal blood supply more than during physiological labour, with shorter intervals for replenishment between contractions. The increased fetal hypoxic stress has well-recognised newborn risks, with plausible longer-term biological effects, as discussed.

The risks for both the woman and her baby will be determined by the infusion rate and resulting uterine activity, and the impacts on uterine blood flow. Clinically, this again emphasises the importance of using the lowest possible effective dosage of synthetic oxytocin, with the least impact on uterine physiology and blood flow. This low-dose approach may also reduce the need for co-interventions, which can add additional risks.

These low doses may also be achievable using pulsed administration, which is more aligned with endogenous oxytocin release in labour and allows time for uterine blood flow restoration between pulses. This is likely to benefit the fetus as well.

It is noteworthy that the models and conclusions presented here, derived from this systematic review data, are in alignment with the data and interpretations published from the 1960s to the 1990s by the notable research group including Fritz and Anna-Riitta Fuchs, M. Yusoff Dawood, Peter Husslein and colleagues. This group established fundamental understandings of oxytocin physiology in labour, including clinical implications. Their work remains very relevant to modern clinicians, and readers are urged to read their research and summaries in their excellent review articles and chapters [81, 230–232].

Finally, authors of this review suggest that the need for synthetic oxytocin administration for labour augmentation could be reduced by supporting activation of endogenous oxytocin for labouring women. Oxytocin release is very sensitive to stressors, even very subtle stressors such as unfamiliar persons and being in an unfamiliar surroundings [233]. In contrast, touch, warmth, and friendly, supportive behaviour from caregivers can promote the progress of labour by optimising the release of oxytocin and the activity of the parasympathetic nervous system. (See Uvnäs-Moberg (2019) for further discussion [1].) Models of care that foster physiological birth, such as continuity of midwifery care and continuous support for women during childbirth, may involve optimisation of oxytocin systems [234, 235].

## Conclusions

This paper has focussed on plasma levels of oxytocin in response to synthetic oxytocin administration in labour, birth or postpartum. The possible impacts on the endogenous oxytocin systems of women and offspring have also been considered, since the oxytocin system is involved not only in the physical processes of labour and birth but also adapts maternal physiology and behaviour through central oxytocin effects.

This data shows that maternal plasma oxytocin levels increase moderately in response to synthetic oxytocin infusions in labour. These levels would not be high enough to cross the maternal blood-brain barrier in biologically significant amounts. Furthermore, there was no evidence of transfer of maternal oxytocin to the fetus across the placenta.

These findings strongly refute the possibility that synthetic oxytocin administered in labour could have direct biological effects on maternal or fetal central oxytocin systems.

However, indirect effects from high doses are very likely. Strengthened uterine contractions, along with reduced uterine blood flow, cause a build-up of lactic acid and low pH in uterine tissues, as described. This causes increased maternal pain and physiological stresses, which are signalled to the brain via SNS pathways that activate central stress responses. Longer-term maternal impacts from stress system activation in labour are biologically plausible. For the fetus, restricted blood flow will cause hypoxia to some extent and increase the risks of fetal compromise. Such adverse effects are more likely to occur at higher infusion rates.

Based on this review and considerations of the findings and implications, maternity care approaches that limit the use of synthetic oxytocin to situations where this intervention will clearly do more good than harm are recommended. Where there are strong clinical indicators, this data supports the most conservative use of synthetic oxytocin at the lowest possible infusion rates.

## Supplementary Information

**Additional file 1.** Search strings for systematic review: Description: Search strings for systematic review: ‘Maternal and newborn plasma oxytocin levels in response to maternal synthetic oxytocin administration during labour, birth and postpartum – a systematic review. Implications for the function of the oxytocinergic system’.

## Data Availability

The dataset supporting the conclusions of this review are included within the article and its additional file.

## Abbreviations

ANS: Autonomic nervous system
CINAHL: Cumulative Index of Nursing and Allied Health Literature
cm: Centimetres of cervical dilation
APGAR: Measure of newborn wellbeing
ELISA: Enzyme- linked immunosorbent assay
EIA: Enzyme-linked immunoassay
Fig: Figure
IU: International units
kg: Kilogram
L: Litre
MCR: Metabolic clearance rate
mg: Milligram
min: Minute
mL: Millilitre
μU: Microunit
μg: Microgram
μM: Micromoles per litre
mU: Milliunit
NICU: Newborn intensive care unit
nM: Nanomoles per litre
pg: Picogram
pH: Measure of acidity
pmol: Picomole
P-LAP: Placental leucine aminopeptidase
pM: Picomoles per litre
PPH: Postpartum haemorrhage
PRISMA: Preferred Reporting Items for Systematic reviews and Meta-Analyses
PSNS: Parasympathetic nervous system
PVN: Paraventricular nucleus
RAGE: Receptor for advanced glycation end- products
RIA: Radioimmunoassay
SSC: Skin-to-skin contact
SNS: Sympathetic nervous system
SON: Supraoptic nucleus
SOT: Synthetic oxytocin
UA: Umbilical artery
UV: Umbilical vein
vs.: Versus (See also Table 3 for SI units and oxytocin conversion)
