# Supplementary material for: Maternal and newborn plasma oxytocin levels in response to maternal synthetic oxytocin administration during labour, birth and postpartum – a systematic review with implications for the function of the oxytocinergic system
Source: BMC Pregnancy Childbirth. 2023 Mar 2;23:137. doi: 10.1186/s12884-022-05221-w (PMC9979579; doi:10.1186/s12884-022-05221-w)
Supplement: Supplementary file 1 — Additional file 1. Search strings for systematic review: Description: Search strings for systematic review: ‘Maternal and newborn plasma oxytocin levels in response to maternal synthetic oxytocin administration during labour, birth and postpartum – a systematic review. Implications for the function of the oxytocinergic system’. [file 12884_2022_5221_MOESM1_ESM.pdf]

File name: Additional file 1

Title: Search strings for systematic review: *Maternal and newborn plasma oxytocin levels in response to maternal synthetic oxytocin administration during labour, birth and postpartum – a systematic review with implications for the function of the oxytocinergic system*

Authors: Sarah Buckley , Kerstin Uvnäs-Moberg , Zada Pajalic , Karolina Luegmair , Anette Ekström-Bergström , Anna Dencker , Claudia Massarotti , Alicja Kotlowska , Leonie Callaway , Sandra Morano , Ibone Olza and Claudia Meier Magistretti

Background:

Configured by Sarah Buckley and Anette Ekstrom-Bergstrom with assistance from librarians at the University of Queensland, Brisbane, Australia and librarians at the University of Skövde, Sweden, respectively. Initial search performed 26 September 2017, repeated March 2020 and June 2022 to include all publications up to June 14 2022.

Terms used:

General and database-specific terms for: oxytocin AND levels AND blood/plasma AND labour/birth/interventions/breastfeeding/newborns

(This <u>review</u> included only those papers within this search with synthetic oxytocin as the intervention)

Pubmed:

((((("Blood/analysis"[Mesh] OR "Blood/blood"[Mesh] OR "Blood/chemistry"[Mesh] OR "Blood/diagnosis"[Mesh] OR "Blood/drug effects"[Mesh] OR "Blood/pathology"[Mesh] OR "Blood/physiopathology"[Mesh] OR "Blood/statistics and numerical data"[Mesh] OR "Blood/toxicity"[Mesh]))) OR ("Diagnostic Techniques and Procedures/blood"[Mesh])) OR "Biological Assay"[Mesh:NoExp]) OR plasma[tiab]) OR "Plasma"[Mesh]) OR circulat*[tiab]) OR blood*[tiab])) AND ((((oxytocin[tiab] OR oxytocin[nm] OR "Oxytocin"[Mesh] OR Pitocin[tiab] OR syntocin*[tiab])) AND (((((Concentration[tiab] OR Level[tiab] OR Amount[tiab] OR Quantity[tiab] OR Sum[tiab] OR Total[tiab] OR RIA[tiab] OR *assay[tiab] OR Measure*[tiab])))))))) AND (("Pregnancy"[Mesh] OR

"Parturition"[Mesh] OR (("Postpartum Period"[Mesh] OR "Obstetric Labor Complications"[Mesh] OR "Delivery, Obstetric"[Mesh] OR "Labor, Obstetric"[Mesh] OR OR "Analgesia, Epidural"[Mesh] OR "Labor, Induced"[Mesh] OR "Fetus"[Mesh] OR "Obstetric Labor Complications"[Mesh] OR "Milk, Human"[Mesh] OR "Breast Feeding"[Mesh] OR "Lactation"[Mesh] OR "Parturition"[Mesh] OR "Delivery, Obstetric"[Mesh] OR intrapartum[Title/Abstract]) OR newborn[Title/Abstract] OR neonat*[Title/Abstract] OR "Infant, Newborn"[Mesh] OR labor[Title/Abstract] OR labour[Title/Abstract] OR caesarean[Title/Abstract] OR cesarean[Title/Abstract] OR birth [Title/Abstract] or obstetric*[Title/Abstract] OR postnatal[Title/Abstract] OR post-natal[Title/Abstract] OR postpartum[Title/Abstract] OR epidural[Title/Abstract]

Scopus

(TITLE-ABS-KEY (oxytocin OR syntocin* OR pitocin)) AND ((TITLE-ABS-KEY (level* OR assay OR ria OR radioimmunoassay OR sum OR total OR measure* OR concentrat* OR amount OR quantity))) AND (TITLE-ABS-KEY (blood W/3 level* OR plasma W/3 level* OR circulat* W/3 level* OR plasma)) AND (TITLE-ABS-KEY (postpart* OR postnatal* OR puerperium OR intrapart* OR perinat* OR labor* OR labour* OR birth* OR childbirth* OR parturit* OR pregnancy OR cesarean* OR caesarean* OR neonat* OR infant* OR fetus OR fetal OR delivery OR epidural OR breastfeed* OR obstetric* OR umbilic* OR (induc* W/3 labo*r) OR (augment* W/3 labo*r) OR forceps OR amniotom* OR "artificial rupture of membranes" OR (synthetic W/3 oxytoci*) OR (breast* W/3 feed*) OR (breast* W/3 lactat*) OR (breast* W/3 milk*) OR (lactat* W/3 milk*) OR breastfeed*))

Cinahl

(TI (pitocin OR syntocin* OR oxytocin) OR AB (pitocin OR syntocin* OR oxytocin) OR MH oxytocin) AND (TI (level OR concentrat* OR amount OR sum OR total OR measure

OR *assay OR quantity OR RIA) OR AB (level OR concentrat* OR amount OR sum OR total OR measure OR *assay OR quantity OR RIA) OR MH (("Biological Assay") OR ("Radioimmunoprecipitation Assay") OR ("Enzyme-Linked Immunosorbent Assay"))) AND (TI (Plasma OR blood* OR circulat*) OR AB (Plasma OR blood* OR circulat*)) AND ((MH childbirth) OR (MH "Postnatal Period+") OR (MH "Pregnancy+") OR TI labo*r OR AB labo*r OR TI post*natal OR AB post*natal OR TI post*partum OR AB post*partum OR TI c*esarean OR AB c*esarean OR TI newborn OR AB newborn OR TI neonat* OR AB neonat* OR TI infant* OR AB infant*)

Psycinfo

((title:(postpart* OR postnatal* OR puerperium OR intrapart* OR perinat* OR labor* OR labour* OR birth*OR childbirth* OR parturit* OR pregnancy OR cesarean* OR caesarean* OR neonat* OR infant* OR fetus OR fetal OR delivery OR epidural OR breastfeed* OR obstetric* OR umbilic* OR forceps OR amniotom* OR "artificial rupture of membranes" OR breastfeed*)) OR (abstract:(postpart* OR postnatal*OR puerperium OR intrapart* OR perinat* OR labor*OR labour* OR birth* OR childbirth* OR parturit* OR pregnancy OR cesarean* OR caesarean* OR neonat*OR infant* OR fetus OR fetal OR delivery OR epidural OR breastfeed* OR obstetric* OR umbilic* OR forceps OR amniotom* OR "artificial rupture of membranes" OR breastfeed*)) OR (Keywords: (postpart* OR postnatal* OR puerperium OR intrapart* OR perinat*OR labor* OR labour OR birth* OR childbirth* OR parturit* OR pregnancy OR cesarean* OR caesarean*OR neonat* OR infant* OR fetus OR fetal OR delivery OR epidural OR breastfeed* OR obstetric* OR umbilic*OR forceps OR amniotom* OR "artificial rupture of membranes" OR breastfeed*))) AND ((title: (blood OR plasma OR circulat*)) OR (abstract: (blood OR plasma OR circulat*)) OR (Keywords: (blood OR plasma OR circulat*))) AND ((title: (level*) OR title: (assay) OR title: (ria) OR title: (radioimmunoassay)OR title: (sum) OR title: (total) OR title: (measure*)OR title:

(concentrat*) OR title: (amount) OR title: (quantity)) OR (Keywords: (level*) OR Keywords: (assay) OR Keywords: (ria) OR Keywords: (radioimmunoassay) OR Keywords: (sum) OR Keywords: (total) OR Keywords: (measure*) OR Keywords: (concentrat*) OR Keywords: (amount) OR Keywords: (quantity)) OR (abstract: (level*) OR abstract: (assay) OR abstract: (ria) OR abstract: (radioimmunoassay) OR abstract: (sum) OR abstract: (total) OR abstract: (measure*) OR abstract: (concentrat*) OR abstract: (amount) OR abstract: (quantity))) AND ((title: (oxytocin) OR title: (Pitocin)OR title: (syntocin*)) OR (Keywords: (oxytocin) OR Keywords: (Pitocin) OR Keywords: (syntocin*)) OR (abstract: (oxytocin) OR abstract: (Pitocin) OR abstract: (syntocin*)))
